# Photoresponsive Polymers for Debonding-on-Demand Pressure-Sensitive Adhesives

**DOI:** 10.1021/acsami.5c25897

**Published:** 2026-03-26

**Authors:** Ibrahim O. Raji, Nishad Dhopatkar, Thomas G. Ribelli, Madison Saldanha, Michele Fromel, Eric Bartholomew, Dominik Konkolewicz

**Affiliations:** a Department of Chemistry and Biochemistry, 6403 Miami University, 651 E High St., Oxford, Ohio 45056, United States; b 93194 Avery Dennison, 171 Draketown Rd, Mill Hall, Pennsylvania 17751, United States

**Keywords:** pressure-sensitive adhesive, coumarin dimerization, debonding, photochemistry, dynamic bonding, responsive networks

## Abstract

An ideal adhesive requires strong adhesion during usage and simple removal on demand, but obtaining these striking features has been a perpetual impediment in the design of pressure-sensitive adhesives (PSAs). To overcome this continuous challenge, a photoresponsive coumarin containing PSA was designed that enables an increase in cross-link density through cycloaddition in the presence of UV light. The PSA was based on either permanent static covalent bonds or dynamic metal ligand coordination bonds. Thermal and rheological experiments revealed the photoinduced cycloaddition, which induced higher cross-link density leading to increased stiffness and ultimately substantially reduced adhesion and debonding. This presented practical evidence to demonstrate debond-on-demand (DoD) adhesion. The lap shear experiment highlights the improved adhesive strength of the PSAs especially for networks with metal ligand dynamic but a persistent cross-linker, while still retaining debonding ability after UV exposure. Considering these results, the simple photoresponsive PSA designed herein demonstrates outstanding potential as a DoD PSA.

## Graphical abstract

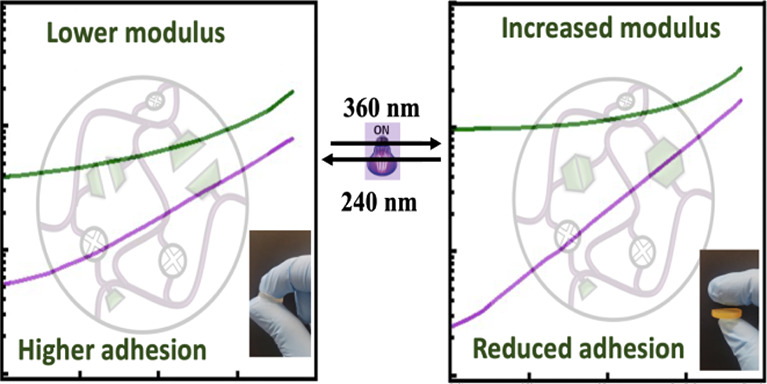

## Introduction

Adhesives are an essential class of polymers that are used extensively to accomplish the important purpose of bonding materials together throughout our daily lives.^1,2^ Pressure-sensitive adhesives (PSAs) are viscoelastic materials that have attracted tremendous attention in the last decades due to their wide range of applications.^1,3^ They are used in many industries from cutting-edge technology such as medical,^4,5^ electronics,^6,7^ and aviation devices^8,9^ to commodity materials for packaging, office, personal, and household items.^1,2,10^ Unlike structural adhesives, which often require chemical processing to activate the bonding, PSAs bond rapidly upon gentle pressure. Bonding two surfaces through a PSA occurs without heat or chemical activation and requires minimal surface preparation.^11,12^ However, to satisfy the requirements of a circular plastics economy for sustainability, well-separated plastics recycling streams are necessary.^13,14^ Products that have a polymer of one type adhered to a polymer or metal of another type, which commonly occurs with a container adhered to a label, represent a unique challenge for separating these two materials. In order to create well-separated waste streams for recycling, adhesive interactions must be broken, either mechanically or chemically. This makes it essential to create pathways to debonding interfaces that could facilitate recycling or reprocessing of previously bonded materials.^15–17^ Developing PSAs with debonding-on-demand (DoD) properties offers a pathway to separating two materials adhered together through a PSA.^16–18^ The development of synthetic PSAs with DoD features has advanced rapidly over the past few years.^3,18–21^ This progress can be attributed to the use of stimuli-responsive^22^ and dynamic covalent^23^ polymers, which offer PSAs with not only enhanced adhesive strength during use but also simple and clean debonding.^21–23^ Stimuli-responsive PSAs undergo physicochemical changes in response to external stimuli like temperature,^20^ voltage,^24^ pH,^25^ or light.^26^

Among these stimuli, the use of light responsive polymers has emerged as a promising strategy in tuning adhesion strengths and inducing potential DoD under ambient conditions. Photoirradiation enables spatiotemporally controlled, direct, contactless, and remote stimulation of materials’ macroscopic properties.^11,12,26^ Interesting findings have been reported in earlier studies, showing the prospects of photoreversible switchable PSAs. The studies by Kim et al. and June et al. showed that *o*-nitrobenzyl-based polymers can undergo photoreversibility under light to decrease the adhesive strength of PSAs.^27,28^ Li and colleagues systematically developed photoswitchable adhesives based on azobenzene networks with high adhesive strength under visible light but low adhesion under UV.^29^ Martella et al. developed polymers containing arylazoisoxazole (AIZ)–azobenzene analogues, which are significantly responsible for photoreversible trans–cis isomerization under irradiation with UV and green light for stronger and reduced adhesion, respectively.^30^ It has been shown by previous reports that incorporating photoreversible coumarin moieties in network materials make them useful in 3D-printing technology,^31^ dynamic photohealing,^32^ shape memory networks,^33^ and PSAs.^11^ Photoinitiated [2 + 2] cycloaddition of coumarin-containing polymer networks has been utilized to modulate the DoD properties of adhesives. For example, Trenor et al. reported the synthesis of copolymers with photo-cross-linkable coumarin moieties, which was introduced through side chain esterification reaction. Their work highlights that peel strength can be reduced via dimerization of the coumarin functionalities upon exposure to UV.^34^ Saito and co-workers developed photoresponsive coumarin-terminated four-arm siloxane polymers, and DoD of the adhesives was achieved through cycloaddition of the coumarin moieties.^11^ Also, Saito and colleagues reported photoswitchable PSAs based on coumarin-functionalized epoxy soybean oil with reduced adhesive strength after photodimerization under UV irradiation.^35^ Although coumarin containing materials have been studied for DoD applications, the majority of the reports involve multistep functionalizing of coumarin moiety via side chain modifications to make block polymers or in many cases the adhesive state of the polymer is an uncross-linked polymer melt, which can be susceptible to long-term stability issues.

Herein, our study describes DoD PSAs based on the incorporation of functional static covalent and dynamic noncovalent photoreversible coumarin into the backbone of the acrylic polymer. The photoresponsive coumarin monomers with polymerizable vinyl units were synthesized and polymerized with a static covalent cross-linker ethylene glycol dimethacrylate (EGDMA) and butyl acrylate (BA) to obtain strong adhesion and light-responsive easy detachment. Furthermore, dynamic metal ligand persistent cross-links based on Al^3+^ binding to carboxylic acids pendant off the polymer backbone with responsive coumarin linkers were synthesized. In the presence of 360 nm UV light, the coumarin moiety undergoes dimerization through [2 + 2] cycloaddition, which increases the cross-link density of the networks, enabling clean and straightforward debonding of bonded parts. This study further provides insights into designing smart and easily synthesized PSAs with strong adhesion while in use and photoresponsive debonding with simple steps, which are in high demand in adhesive and coatings industries for sustainability.

## Results and Discussion

The copolymers were synthesized using free radical polymerization, as shown in Scheme 1. The copolymer PSAs consist of butyl acrylate (BA), which serves as the backbone of the chain, ethylene glycol dimethacrylate as the static cross-linker, and photoresponsive 2-oxo-2*H*-chromen-7-yl acrylate (coumarin monomer (CM)). For the dynamic system, EGDMA was substituted with metal chelate aluminum acetyl acetonate and acrylic acid (Al-AA). The low-temperature thermal initiator 2,2’-azobis­(4-methoxy-2,4-dimethylvaleronitrile) (V-70) was used. In Scheme 1, the excellent efficiency of coumarin dimerization is represented with the bold solid arrow at 360 nm, while the reverse reaction can be less efficient and is represented by the dashed arrow at 250 nm.

**Scheme 1:**
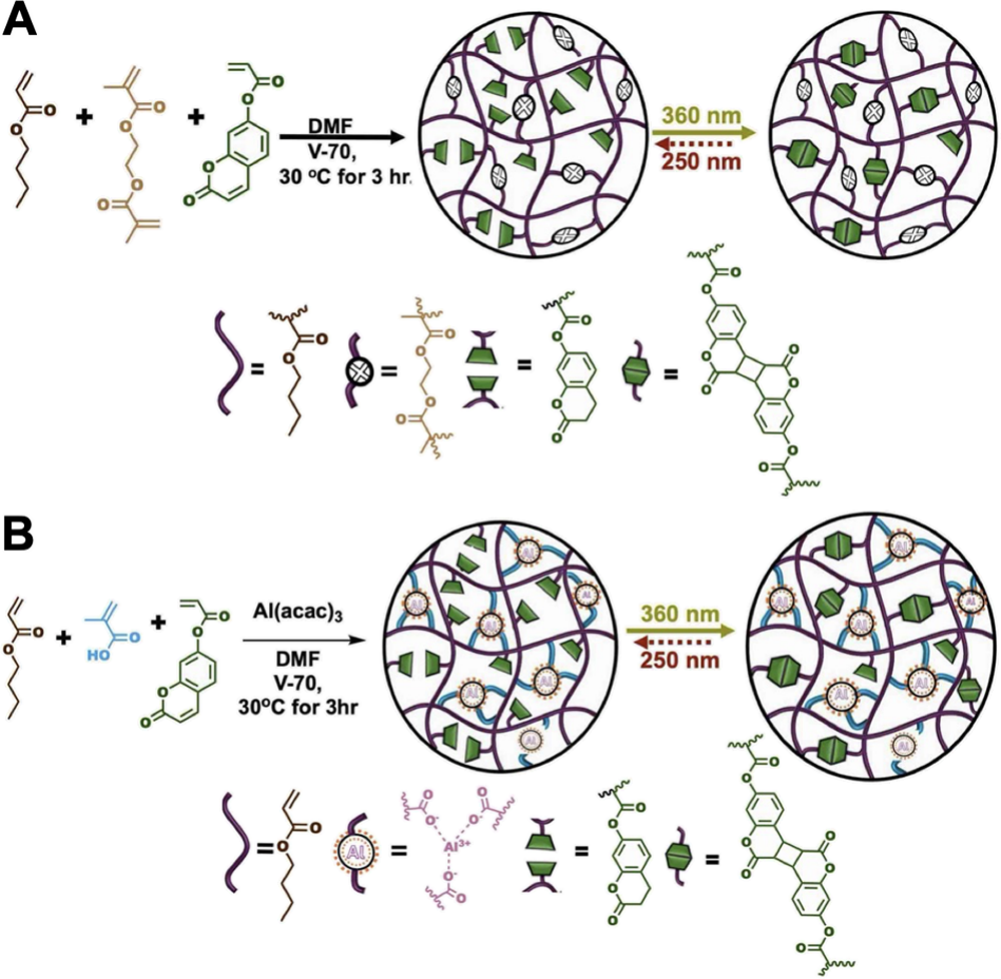
Synthetic Scheme for (a) Nondynamic EGDMA-Based Network and (b) Al-AA-Based Dynamically Cross-Linked Network^a^

a Reactions under 360 nm led to debonding, while those under 250 nm led to rebonding. Efficiency of the efficiency of dimerization is higher than the reverse reaction as indicated by the dashed (250 nm) vs bold arrow (360 nm).

The polymers synthesized are poly­(BA-EGDMA-CM) and poly­(BA-Al-AA-CM) with varied cross-link density. In all cases, conventional free radical polymerization was used, with the poly­(BA-EGDMA-CM) and poly­(BA-Al-AA-CM) reporting the wt % of each unit within the polymer. Using the static EGDMA linker gave poly­(BA_94.5_-EGDMA_0.5_-CM_5_), poly­(BA_89.5_-EGDMA_0.5_-CM_10_), poly­(BA_94_-EGDMA_1_-CM_5_), and poly­(BA_89_-EGDMA_1_-CM_10_). The density of the dynamic Al-AA cross-linker was varied to give poly­(BA_91.5_-Al_0.5_-AA_3_-CM_5_), poly­(BA_86.5_-Al_0.5_-AA_3_-CM_10_), poly­(BA_88_-Al_1_-AA_6_-CM_5_), and poly­(BA_83_-Al_1_-AA_6_-CM_10_). *n*-Dodecylmercaptan (0.5%, DDM) was added to the Al-AA-based polymers to modulate the molecular weight, since in the absence of DDM macroscopic gelation occurred preventing the addition of Al­(acac)-based cross-linker. For each of the coumarin-based PSAs, the swelling ratio and gelation content were determined. In each case, the swelling ratio decreased upon exposure to 360 nm UV radiation. The effect is substantially more pronounced, i.e., a larger reduction in swelling ratio is measured, in systems that contained 10% CM, with a smaller effect in systems with just 5% CM. This is consistent with the higher CM content leading to more cross-links upon UV irradiation. Furthermore, the gelation content of the PSAs was carried out, and all the PSAs have a gel content above ∼95% before 360 nm UV irradiation and above 94% after 360 nm UV irradiation (Table S1), which indicates essentially complete gelation, with minimal difference in net gel content, since each PSA already had a very high gel content, even before coumarin dimerization.

Initially, photorheometry was performed to verify the photoinduced nature of the dimerization. As shown in Figure 1, for both the dynamic and static cross-linked networks, cycloaddition of the coumarin moiety occurred after ∼200 s, which corresponds to the time when the long-wave UV irradiation was activated (UV-A). Two light intensities were used to investigate the photoresponsiveness of the networks, labeled L for low intensity (57 mW/cm^2^) and H for high intensity (291 mW/cm^2^). With the higher (H) light intensity, cycloaddition occurs faster especially in the networks with 10% CM monomer compared to lower light intensity of (L) and lower percentage of CM. Higher *G*′ moduli increases between ∼20,000 and 90,000 Pa were achieved for networks (both dynamic and nondynamic) with 10% CM monomer, while ∼1200–11,000 Pa were achieved for networks with 5% CM monomer (Figure S1). This difference reveals the importance of having relatively higher CM content for a higher *G*′ increase due to the higher density of cross-linkers formed after irradiation.^36^ This is consistent with the higher CM loading leading to more potential UV induced cross-linking sites through cycloaddition. Additionally, the dynamic Al-AA-based materials have higher mobility, potentially enabling more effective cross-linking through better segmental motion than the static EGDMA system. When there is more CM along with the dynamic Al-AA linker, this leads to the greatest increase in storage modulus upon photoirradiation due to improved chain mobility from Al-AA and more potential UV-induced cross-links from more CM units.

**Figure 1:**
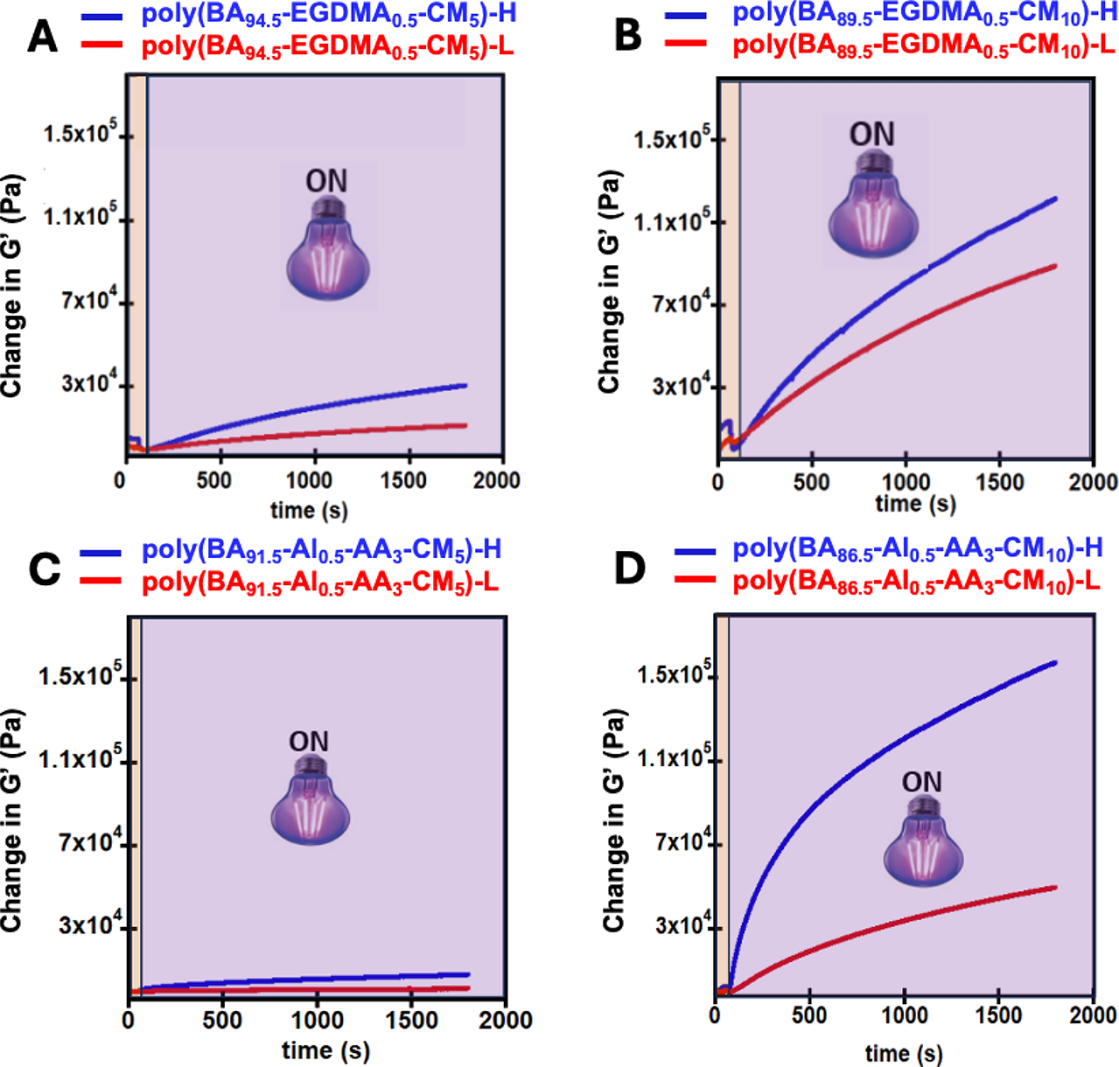
Photorheometry time sweeps of the light-responsive PSAs for (a) poly­(BA_94.5_-EGDMA_0.5_-CM_5_), (b) poly­(BA_84.5_-EGDMA_0.5_-CM_10_), (c) poly­(BA_91.5_-Al_0.5_-AA_3_-CM_5_), and (d) poly­(BA_86.5_-Al_0.5_-AA_3_-CM_10_). L refers to the low intensity (57 mW/cm^2^) UV-A, and H for thehigh intensity (291 mW/cm^2^) UV-A.

Differential scanning calorimetry (DSC) was used to investigate the thermal behavior of the coumarin-containing PSAs. Measuring *T*_g_ is an indirect measure of a material’s ability to function as a PSA. If a material’s *T*_g_ increases due to applied stimulus, the adhesive properties should decrease, due to reduced chain mobility.^37^ PSAs typically have a *T*_g_ between −60 and −10 °C,^38^ and as Table 1 and Figures 2 and 3 illustrate, all of the samples’ *T*_g_ fall within the range needed for typical PSAs. Typical DSC curves before and after UV irradiation are shown in Figure 2 for the BA-Al-AA-CM systems and in Figure 3 for the BA-EGDMA-CM systems. The averages of the second and third runs are given with standard deviation in Table 1 both before and after irradiation with UV. The *T*_g_ of the dual cross-linked coumarin-containing PSAs was tuned by exposing to UV irradiation of a wavelength (λ) of ∼360 nm. Upon exposure to light with an intensity of 4.8 ± 0.5 mW/cm^2^, all of the coumarin-containing PSAs (especially the dynamic cross-linked networks) showed an increase in the *T*_g_, consistent with an increase in the cross-link density of the networks. The additional cross-links are attributed to dimerization through 2 + 2 cycloaddition of the coumarin moiety,^31,39^ consistent with the results of the photorheometry study.

**Figure 2:**
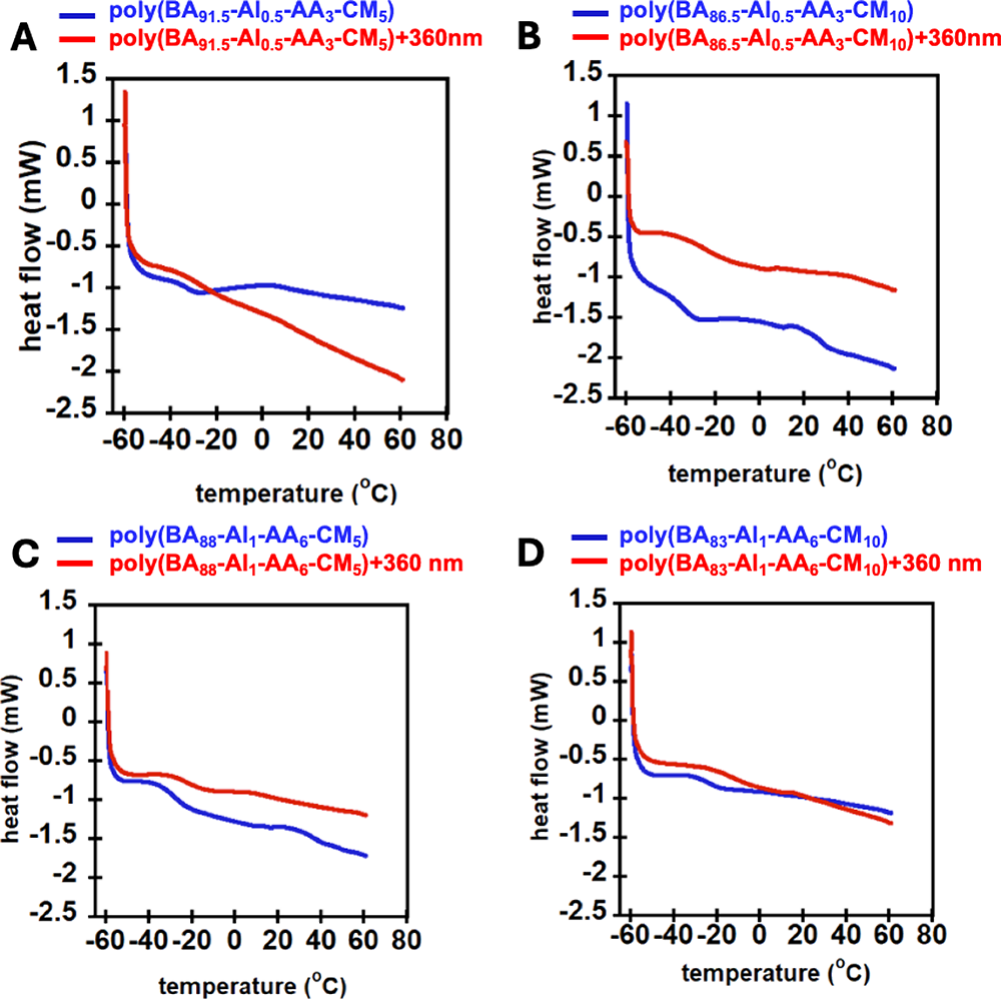
DSC plots before and after 360 nm UV for (a) poly­(BA_91.5_-Al_0.5_-AA_3_-CM_5_), (b) poly­(BA_86.5_-Al_0.5_-AA_3_-CM_10_), (c) poly­(BA_88_-Al_1_-AA_6_-CM_5_), and (d) poly­(BA_83_-Al_1_-AA_6_-CM_10_).

**Table 1:** Table of Synthesized Networks^a^

networks	*T*_g_ (°C)^a^	*G*′ (kPa)^b^	*S*_shear_ (PET) (kPa)^c^	volume reduction (%)^d^
poly(BA_91.5_-Al_0.5_-AA_3_-CM_5_)	–32.4 ± 0.3	5.2	22 ± 3	
poly(BA_91.5_-Al_0.5_-AA_3_-CM_5_) + 360 nm	–24 ± 1	10.9		3 ± 1
poly(BA_86.5_-Al_0.5_-AA_3_-CM_10_)	–35 ± 2	8.3	45 ± 1	
poly(BA_86.5_-Al_0.5_-AA_3_-CM_10_) + 360 nm	–23 ± 1	28.4		10.5 ± 0.9
poly(BA_88_-Al_1_-AA_6_-CM_5_)	–27.1 ± 0.5	10.6	44 ± 10	
poly(BA_88_-Al_1_-AA_6_-CM_5_) + 360 nm	–21.4 ± 0.4	18.6		6 ± 1
poly(BA_83_-Al_1_-AA_6_-CM_10_)	22.7 ± 0.03	15.5	51 ± 2	
poly(BA_83_-Al_1_-AA_6_-CM_10_) + 360 nm	–13 ± 2	36.1		10 ± 4
poly(BA_94.5_-EGDMA_0.5_-CM_5_)	–38.7 ± 0.5	8.3	24 ± 4	
poly(BA_94.5_-EGDMA_0.5_-CM_5_) + 360 nm	–33.5 ± 0.8	20.7		5.2 ± 0.6
poly(BA_89.5_-EGDMA_0.5_-CM_10_)	–37.8 ± 0.3	15.1	10 ± 3	
poly(BA_89.5_-EGDMA_0.5_-CM_10_) + 360 nm	–30.8 ± 0.3	37.2		12 ± 4
poly(BA_94_-EGDMA_1_-CM_5_)	–33.3 ± 0.5	14.1	17 ± 4	
poly(BA_94_-EGDMA_1_-CM_5_) + 360 nm	–31 ± 2	24.9		9 ± 3
poly(BA_89_-EGDMA_1_-CM_10_)	–37.5 ± 0.2	18.8	20 ± 5	
poly(BA_89_-EGDMA_1_-CM_10_) + 360 nm	–25.8 ± 0.9	43.9		17 ± 5

a (a) *T*_g_ before and after 360 nm UV exposure, (b) storage modulus measured at 1 rad/s at 25 °C before and after 360 nm UV exposure, (c) adhesive strength measured by lap shear with PET substrate, and (d) volume reduction after 360 nm UV exposure.

**Figure 3:**
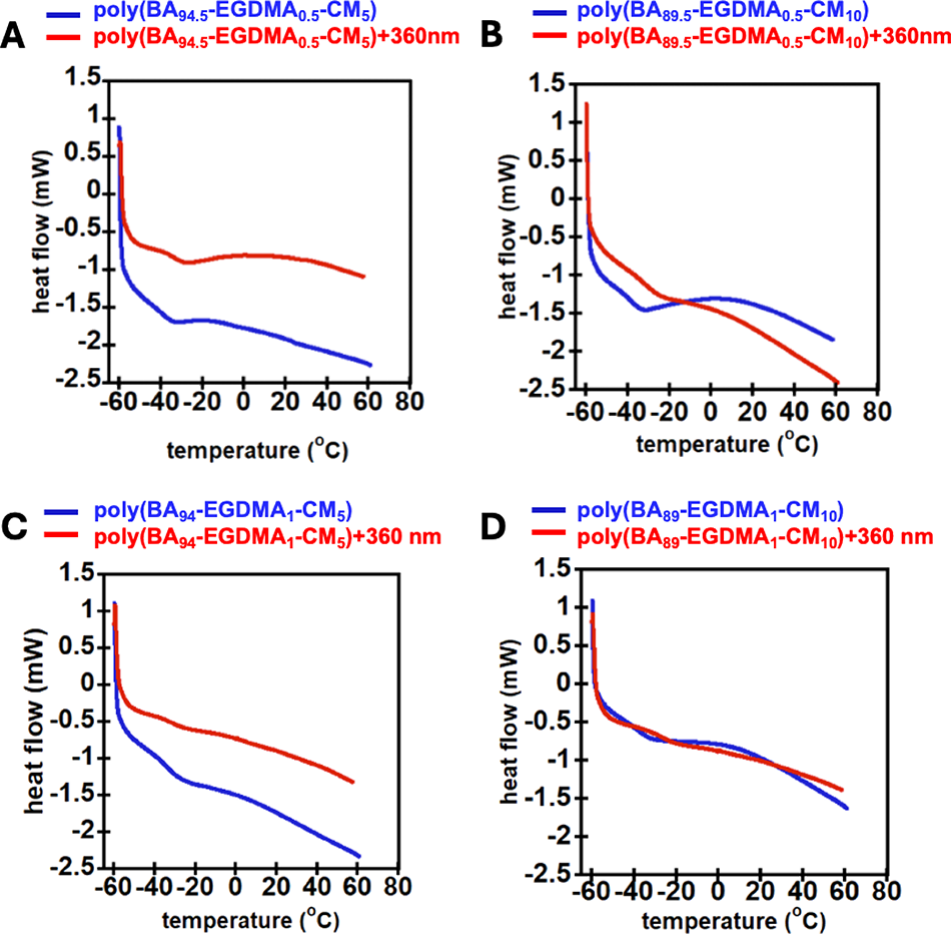
DSC plots before and after 360 nm UV irradiation for (A) poly­(BA_94.5_-EGDMA_0.5_-CM_5_), (B) poly­(BA_89.5_-EGDMA_0.5_-CM_10_), (C) poly­(BA_94_-EGDMA_1_-CM_5_), and (D) poly­(BA_89_-EGDMA_1_-CM_10_).

The photoinduced increase in cross-link density of the networks raised the *T*_g_, consistent with previous studies reported in the literature on the influence of coumarin dimerization.^33,39^ As shown in Figures 2 and 3 and Table 1, after UV irradiation, networks with 10% coumarin had a larger increase in *T*_g_ compared to materials with a 5% coumarin moiety. This is expected because the increase in cross-link density is directly correlated with the coumarin monomer required for dimerization.^39^ Interestingly, the dynamic Al-AA-based linker showed notably higher increases in *T*_g_ by ∼9 °C after UV irradiation compared to the nondynamic EGDMA cross-linked materials, which increased by ∼7 °C on average. In all cases, the increases in *T*_g_ are substantially larger than the variability in the *T*_g_ measurement reported in Table 1. The large increase in *T*_g_ of the photoresponsive PSAs indicates that UV-driven cross-linking occurs throughout the bulk of the materials, potentially enabling DoD properties.^11^ Furthermore, FTIR studies were performed on the coumarin-based PSAs. FTIR scans were taken before and after UV irradiation to examine the dimerization of the coumarin moiety. As shown in Figure S5, the peak around 1600–1650 cm^–1^ (which is the vinyl group responsible for dimerization significantly) reduces upon 360 nm UV irradiation. This shows that upon light exposure, the vinyl group undergoes [2 + 2] cycloaddition, and the peak tends to reduce accordingly.

Complementary to the thermal investigation, rheological analysis giving viscous (*G*″) and elastic (*G*′) responses change with network composition, frequency, or in this case, UV irradiation. In general, PSAs should have rheological moduli below 100 kPa at room temperature and 1 Hz, according to the Dahlquist criterion.^40^ The materials have moduli of about 5–50 kPa before UV irradiation, which satisfies Dahlquist criterion. Materials with moduli below 100 kPa give superior adhesion to a substrate and allow for proper wetting of the substrate. In contrast, a modulus that is too low may lack shear resistance, which can happen below 5 kPa. Figure 4A–D shows frequency sweep plots before and after exposure to 360 nm UV irradiation for the EGDMA-CM cross-linked networks, and Figure 5A–D shows frequency sweep plots before and after UV irradiation of 360 nm for the dynamic Al-AA-CM network. All of the samples exhibit typical viscoelastic solid behavior, as shown in Figures 4 and 5, where the storage modulus (*G*′) is higher than the loss modulus (*G*″) at low frequencies and transitions toward a glass transition-like behavior at higher frequencies. Within a rubbery-like plateau, the cross-link density is directly proportional to the observed value of *G*′, according to the theory of rubber elasticity.^41^

**Figure 4:**
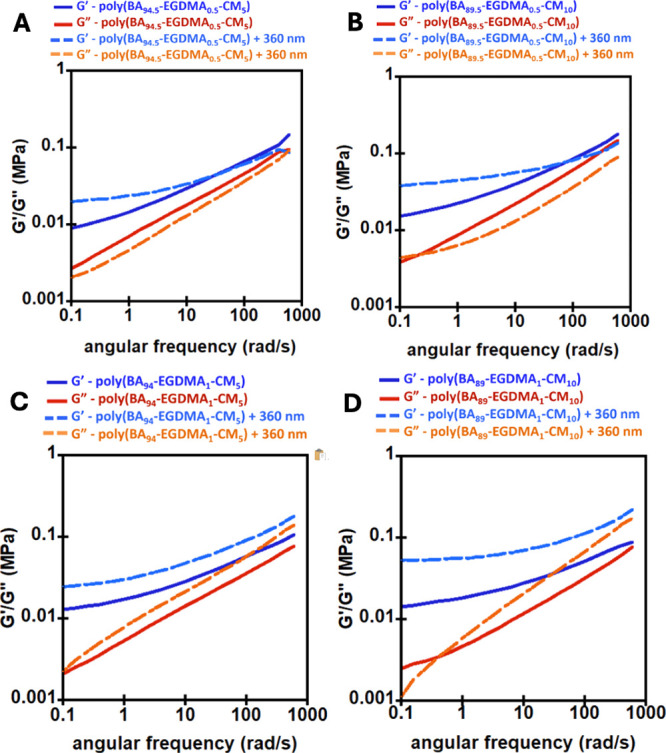
Frequency sweep plots before and after 360 nm UV for (A) poly­(BA_94.5_-EGDMA_0.5_-CM_5_), (B) poly­(BA_89.5_-EGDMA_0.5_-CM_10_), (C) poly­(BA_94_-EGDMA_1_-CM_5_), and (D) poly­(BA_89_-EGDMA_1_-CM_10_).

**Figure 5:**
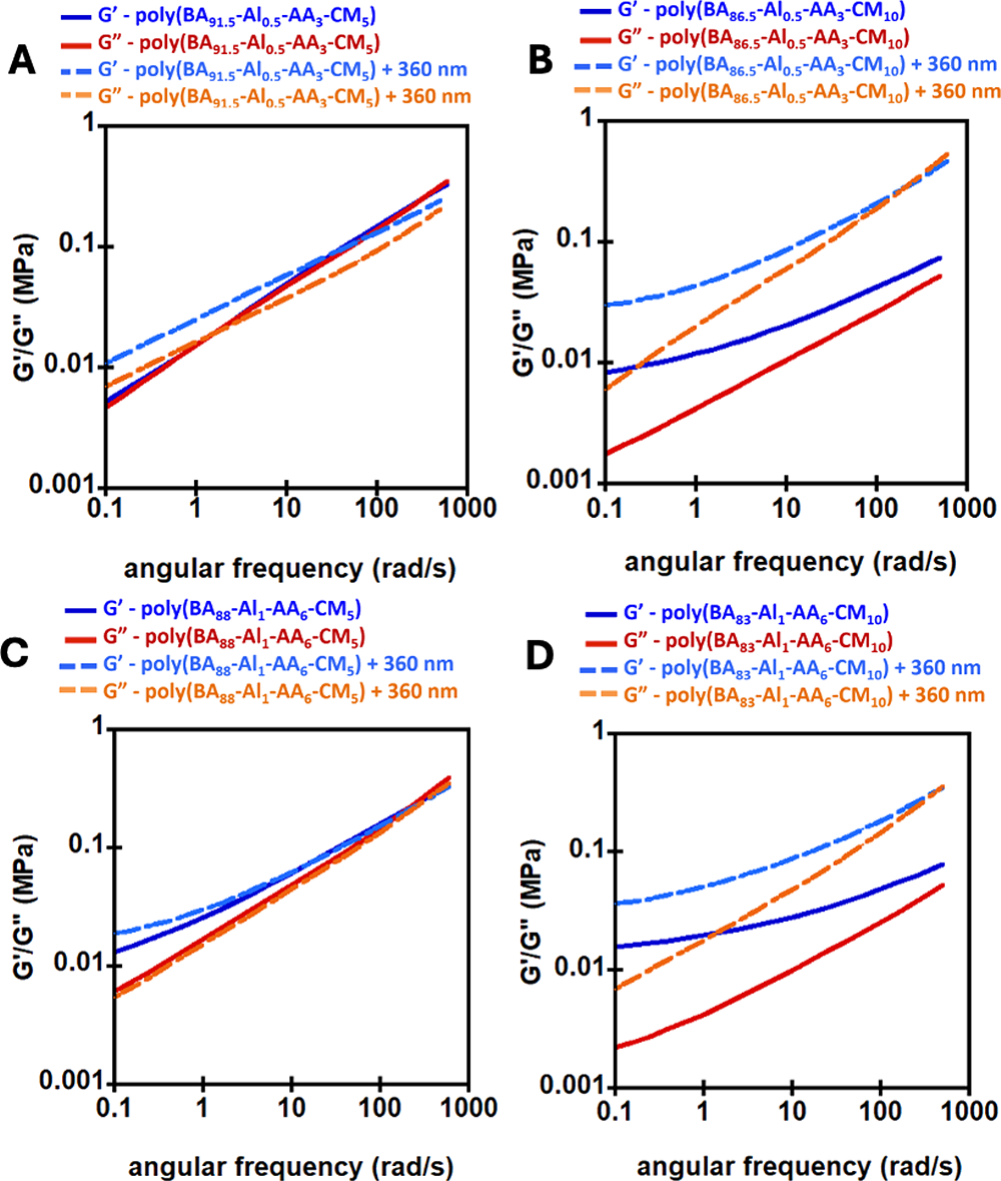
Frequency sweep plots before and after 360 nm UV for (A) poly­(BA_91.5_-Al_0.5_-AA_3_-CM_5_), (B) poly­(BA_86.5_-Al_0.5_-AA_3_-CM_10_), (C) poly­(BA_88_-Al_1_-AA_6_-CM_5_), and (D) poly­(BA_83_-Al_1_-AA_6_-CM_10_).

As seen in Figure 4, the EGDMA cross-linked networks all showed a notable increase in the storage modulus *G*′ after UV irradiation. Materials containing 5% CM showed a relatively smaller increase in *G*′ after UV than networks containing 10% CM, as anticipated by the density of potential cross-links in the material. Figure S2 shows the frequency sweep plot of EGDMA-CM networks with 1.25% and 2.5% before and after light irradiation. With these very low CM loadings, there was no significant change in moduli after UV exposure. Interestingly, Figure 4B,D shows that the plateau modulus of poly­(BA_89.5_-EGDMA_0.5_-CM_10_) was ∼100 kPa, while the material that contained more static cross-links, poly­(BA_89_-EGDMA_1_-CM_10_), reached a plateau modulus of ∼80 kPa. This indicates that the material with the higher potential for cross-linking (1% EGDMA, 10% CM) reached a lower total effective cross-link density after UV irradiation than the materials with a lower density of permanent cross-links (0.5% EGDMA, 10% CM). This is most likely due to the higher density of permanent EGDMA cross-links restricting chain mobility and limiting the formation of new CM-based cross-links upon UV irradiation. A similar phenomenon was observed with 5% CM-based networks as seen in Figure 4A,C.

As expected, with Al-AA-CM cross-linked polymers in Figure 5, the apparent storage moduli of the materials containing 1% Al and 6% AA were somewhat higher than those with 0.5% Al and 3% AA. This corresponds with rubber elasticity, which means that as the number of cross-links per unit volume increases, the material becomes stiffer and more resistant to deformation, hence increased modulus.^42^ Importantly, in the Al-AA-CM cross-linked polymers, networks with 5% CM showed a smaller increase in *G*′ after UV irradiation compared to networks cross-linked with 10% CM. This is expected because the loading of CM monomer will have significant effect on the cross-link density due to dimerization.^33,39^ The increase in storage moduli, approaching the end of the Dahlquist criterion upon UV irradiation, makes these networks promising for DoD PSAs.

Facile adhesion with the DoD induced by efficient external stimuli presents an ongoing challenge in PSA design. Considering the photoresponsive and dynamic behavior of the PSAs synthesized, the adhesive strength was examined by using lap shear analysis against both poly­(ethylene terephthalate) (PET) and stainless steel (SS). Lap shear measures the cohesive strength of PSAs, which can be influenced by their adhesive interactions with the substrate.

Both the Al-AA-CM- and EGDMA-CM-based materials functioned as PSA materials by lap shear analysis prior to UV irradiation. Figure 6 shows that better adhesive strength (Sshear) was observed with PET than SS in general for both Al-AA-CM- and EGDMA-CM-based materials. BA-based hydrophobic PSAs can exhibit higher adhesion to PET than to stainless steel primarily due to the lower surface energy of PET.^43,44^ Both PET and acrylic PSAs have a dominant dispersive component in their respective surface energies,^43–46^ whereas for stainless steel, the surface energy is dominated by the polar component.^47,48^ This similarity in the dispersive surface energy components (PET ∼30–35 mN/m and p­(BA) ∼30 mN/m) allows better wetting of PET by hydrophobic BA-based PSAs than that over hydrophilic stainless steel.^49,50^ Additionally, the amorphicity in PET allows for microscopic flow of PSA polymer chains into the PET substrate, forming more intimate interfacial contact, further improving the wetting and bonding.^45,49^ Meanwhile, a crystalline, polar stainless steel substrate limits the extent of interface formation with a hydrophobic PSA.^48^ The only exception was poly­(BA_89_-EGDMA_1_-CM_10_) in Figure 6C, which had essentially no adhesion to SS, although it did adhere to PET. Figure 6B shows that the adhesive strength of the Al-AA-CM networks (∼50 kPa) was determined to be the highest, compared to EGDMA-CM networks with ∼25 kPa (Figure 6A). This indicates that the dynamic networks where chain mobility is dominant improve adhesion of the PSAs compared to less dynamic networks with restricted chain mobility. This is likely due to the more dynamic linkers allowing better wetting and compliance of the PSA with the substrate. The stability of the PSAs in aqueous solvent was investigated to determine the effect of water on the adhesion properties of the coumarin-based PSAs. The water stability experiment was carried out by submerging each of the static cross-linked and dynamic cross-linked PSAs inside water for 48 h. After 48 h, the PSAs were pat dried, and their adhesive strengths were determined using a lap shear experiment. The EGDMA-cross-linked PSAs showed great water stability with the adhesive strength (24 ± 4 kPa) before water submersion being quite similar to the adhesive strength (25 ± 0.5 kPa) after water submersion. However, the adhesive strength of Al-AA cross-linked PSAs showed a significant decrease (from 22 ± 3 kPa to 13 ± 3 kPa) upon submersion into water after 48 h (Table S4). The reduced adhesion in Al-AA cross-linked PSAs could be as a result of the possibility of leaching of the metal complexes in aqueous media, which has also been reported in previous works.^51–53^ In addition to PET and SS substrates, adhesion experiments were done on both the EDGMA and Al-AA cross-linked PSAs using a transparent substrate like glass. It is important to note that at and near 360 nm, borosilicate glass is essentially transparent down to ∼320 nm.^54^ Both systems showed quite significant adhesion on a glass substrate with 8 ± 0.5 kPa and 5 ± 0.7 for EGDMA and Al-AA cross-linked networks, respectively (Table S5). This result shows that the coumarin based PSAs synthesized in this work can not only adhere firmly to opaque (SS) substrates, but they can also be used in transparent (glass and PET) substrates. Overall, the mode of failure for the poly­(BA-Al-AA-CM) PSAs is mostly cohesive on either PET or SS substrate. Also, the EGDMA-based system mostly showed adhesive failure for both the PET and SS substrates (Figure S5). Furthermore, after irradiation with 360 nm UV irradiation, the failure mode is adhesive which is a good indication that supports the 100% debonding efficiency.

**Figure 6:**
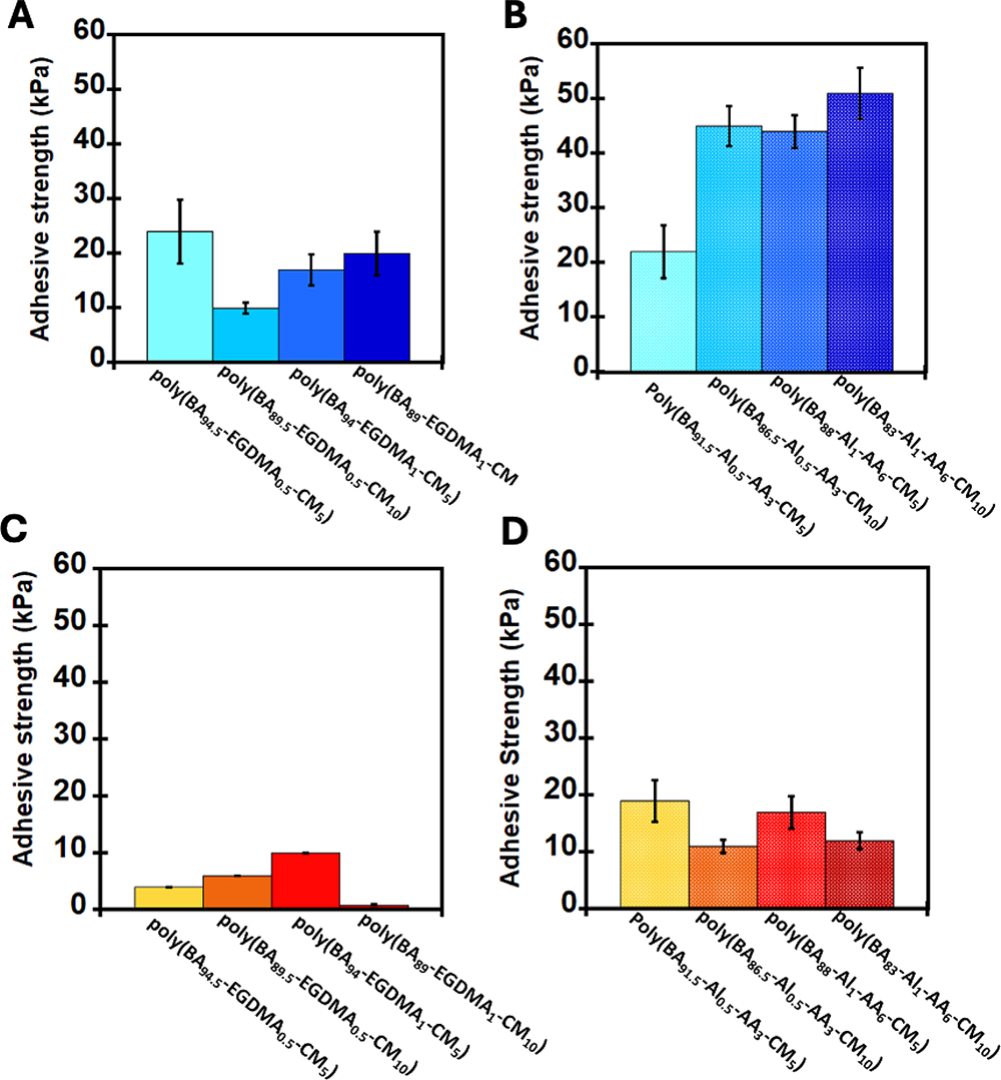
Adhesive test plots for (A) nondynamic cross-linked networks with PET substrate, (B) dynamic cross-linked network with PET substrate, (C) nondynamic cross-linked networks with stainless steel substrate, and (D) dynamic cross-linked networks with stainless steel substrate.

In addition to lap shear, adhesive peels and dynamic shear were done to understand the adhesive properties of the coumarin-based PSAs. For poly­(BA_91.5_-Al_0.5_-AA_3_-CM_5_), adhesion on the PET panel was higher than that on the SS panel. As described previously, the hydrophobic nature of the PSA, the similarity in the dispersive components of the surface energies between PSA and PET, and the amorphous nature of PET panel allow better bonding and hence adhesion on PET than that on SS. With increasing UV intensity, it was clearly observed that the peel adhesion reduced consistently regardless of the substrate. This reduction in peel is due to cross-linking of CM moieties resulting in increased *G*′ reduced free volume and reduced ability to viscoelastically dissipate the deforming forces during the peel test. This effect of peel adhesion reduction upon UV exposure is seen ever more dramatically for 10% CM containing PSAs. In the pristine (unexposed state), the difference in adhesion on the PET panel and SS panel is further amplified for the 10% CM containing PSA. More interestingly, the drop in peel adhesion when exposed to the same UV intensity was more pronounced in the 10% CM PSA than that for the 5% CM. At the highest dose of 7700 mJ/cm^2^ of 360 nm UV irradiation, the peel adhesion was reduced to zero, thus highlighting the debondable nature of the 10% CM containing PSAs.

For both 5% and 10% CM containing PSAs, however, the dynamic shears remained unchanged or changed ever so slightly, regardless of the substrate panel type. This is not surprising considering that the dynamic shear test characterizes the cohesive nature of the PSA, which reflects its elasticity. With UV exposure, there occurs an increase in the cross-linking consuming the CM species and buildup of elasticity, i.e., *G*′ as also seen in our rheology data. Within the confines on the dynamic shear test geometry, this characterization seems not as sensitive to reflect the changes in the elasticity buildup due to CM cross-linking. This observation also teaches an important finding that debonding is subjected to deformation geometry or direction. For our system, debonding is readily possible in peel direction than in shear direction or force fields. This ability of debonding directionality or anisotropy can have some creative practical implications.

Furthermore, to determine the DoD properties of the networks, the photoresponsive PSAs were sandwiched and bonded between two PET substrates and exposed to 360 nm UV irradiation with an intensity of 4.8 ± 0.5 mW/cm2 for 12 h as shown in the setup in Figure 7. Due to optical density, this debonding was only performed on the transparent PET substrate. Figure 7A shows the control setup of the PSA, which lacks the photoresponsive CM unit before and after UV irradiation. As expected, after UV irradiation, no significant decrease in adhesive strength was observed in the standard PSA without coumarin. This is due to the absence of the coumarin monomer, which is responsible for the photoresponsive property of the DoD PSAs. Figure 7B shows the standard PSA with coumarin monomer (poly­(BA89.5-EGDMA0.5-CM10)). Notably, after UV irradiation, an essentially complete decrease of adhesion was observed, leading to the debonding of the PET substrate from the adhesive. This decrease in adhesive strength is also below the detection force of the load cell. This indicates that irrespective of the nature of the persistent cross-linker used, either EGDMA or Al-AA, the coumarin moiety can still undergo dimerization, which increases the cross-link density of the network, facilitating debonding. However, coupled with this increase in cross-link density, the volume of the material decreases, which can further facilitate debonding by creating ruptures in the adhesion. Although there is some yellowing of the samples upon exposure to 360 nm UV radiation, however, as seen by rheology and DSC, the bulk properties, not just the surface properties, are impacted by the UV treatment, and the contracted sample retains its shape as a rectangular prism even after contraction.

**Figure 7:**
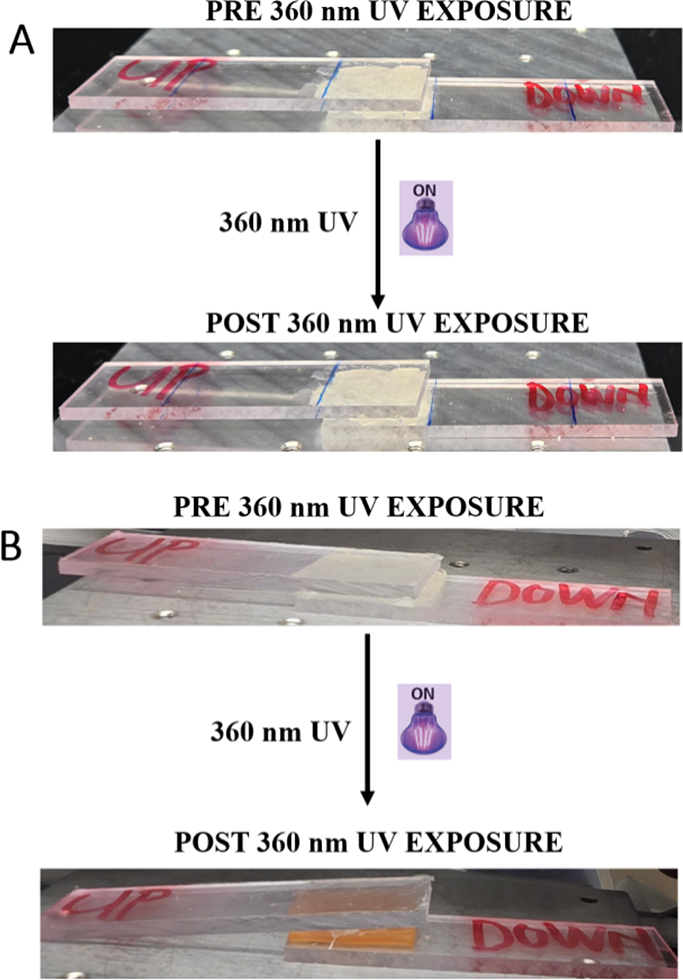
Typical setup for the DoD experiment of the photoresponsive PSAs for (a) control setup without photoresponsive coumarin monomer (poly­(BA_99.5_-EGDMA_0.5_)) and (b) PSA with photoresponsive coumarin monomer (poly­(BA_89.5_-EGDMA_0.5_-CM_10_)) before and after 360 nm UV irradiation for 12 h.

Furthermore, unlike the debonding with an efficiency of >99% (from 24 ± 4 to 0 kPa), the rebonding under 250 nm UV irradiation has a much lower efficiency of about 25% (from 24 ± 4 kPa to 6 ± 1 kPa) in Figure S4 and Table S3, consistent with Scheme 1. Rheological studies confirmed that significant moduli increase upon exposure to 360 nm UV irradiation (Figures 4 and 5), but upon 250 nm UV irradiation, there is an obvious moduli changes but not a complete return to the original value (Figure S4). Figure S7 shows the disappearance of the infrared (IR) band at 1620 cm^–1^ after 360 nm irradiation, attributed to the alkene of the coumarin unit, and some recovery of this band at 1620 cm^–1^ after irradiation with 250 nm. This is consistent with the proposed mechanism in Scheme 1. However, the debonding is substantially more efficient than the rebonding as seen across all these IR, rheological, and adhesive data.

As evident in the rheological and shear data, the PSAs can be efficiently debonded from the substrate upon 360 nm UV irradiation but a little less efficient to regain the adhesive strength upon exposure to 250 nm UV irradiation. However, it is important to note that there is a decrease in the exposure dose that arrive on the coumarin-based PSA when the light is irradiated through the PET substrate. Hence, the amount of light intensity that would eventually arrive on the PSAs’ surface to trigger the DoD is 1.9 mW/cm^2^. Apparently, there’s a reduction in light intensity (from 4.8 mW/cm^2^ to 1.9 mW/cm^2^) that hits the PSA’s surface to trigger the DoD, but it is quite interesting that despite the intensity reduction, there’s an efficient debonding. This indicates sensitivity of the coumarin-based PSAs and that the amount of light that hits the surface of the PSAs when it is been sandwiched in between substrates is sufficient for the dimerization.

The absence of adhesion after 360 nm UV irradiation was observed for all the networks with 5% and 10% CM stipulating the responsiveness and suitable position of the coumarin monomer in the network. Overall, this result correlates with the thermal and rheological experiment highlighting the excellent photoresponsiveness of the PSAs under light resulting to increased cross-link density and ultimately leading to clean and simple debonding of the PSAs. The reduction in bonding efficiency can be correlated with the loss of coumarin alkene signal, consistent with dimerization of coumarin. This is consistently seen in Figure S6, where all samples showed a reduction in alkene signal upon irradiation with 360 nm UV irradiation. The recovery after UV irradiation was less efficient, although measurable in one of the best performing systems (poly­(BA_89.5_-EGDMA_0.5_-CM_10_)). It is important to note that a control PSA with no CM present at all showed no IR signals in a range of 1650–1500 cm^–1^, indicating that the signal 1620 cm^–1^ can be attributed to the coumarin C=C stretch, with its loss upon 360 nm irradiation consistent with dimerization of coumarin.

In addition to lap shear, adhesive peels and dynamic shear were performed to understand the adhesive properties of the coumarin-based PSAs. For poly­(BA_91.5_-Al_0.5_-AA_3_-CM_5_), adhesion on the PET panel was higher than that on the SS panel (Figure 8 and Table S6). Due to the fact that peel and dynamic shear-type experiments use only one rigid substrate (PET/SS) with a flexible PET film (facestock) on the other side of the adhesive, both SS and PET substrates could be used. As described previously, the hydrophobic nature of the PSA, the similarity in the dispersive components of the surface energies between PSA and PET, and the amorphous nature of PET panel allow better bonding and hence adhesion on PET than that on SS. With increasing UV intensity, it was clearly observed that the peel adhesion reduced consistently regardless of the substrate as shown in Figure 8A,B. This reduction in peel is due to cross-linking of CM moieties resulting in increased *G*′, reduced free volume, and reduced ability to viscoelastically dissipate the deforming forces during the peel test. This effect of peel adhesion reduction upon UV exposure is seen ever more dramatically for 10% CM containing PSAs (Figure 8B and Table S6). In the pristine (unexposed state), the difference in adhesion on the PET panel and SS panel is further amplified for the 10% CM containing PSA. More interestingly, the drop in peel adhesion when exposed to the same UV intensity was more pronounced in the 10% CM PSA than that for the 5% CM. In Figure 8B, at the highest dose of 7700 mJ/cm^2^ of 360 nm UV, the peel adhesion was reduced to zero, thus highlighting the debondable nature of the 10% CM containing PSAs.

**Figure 8:**
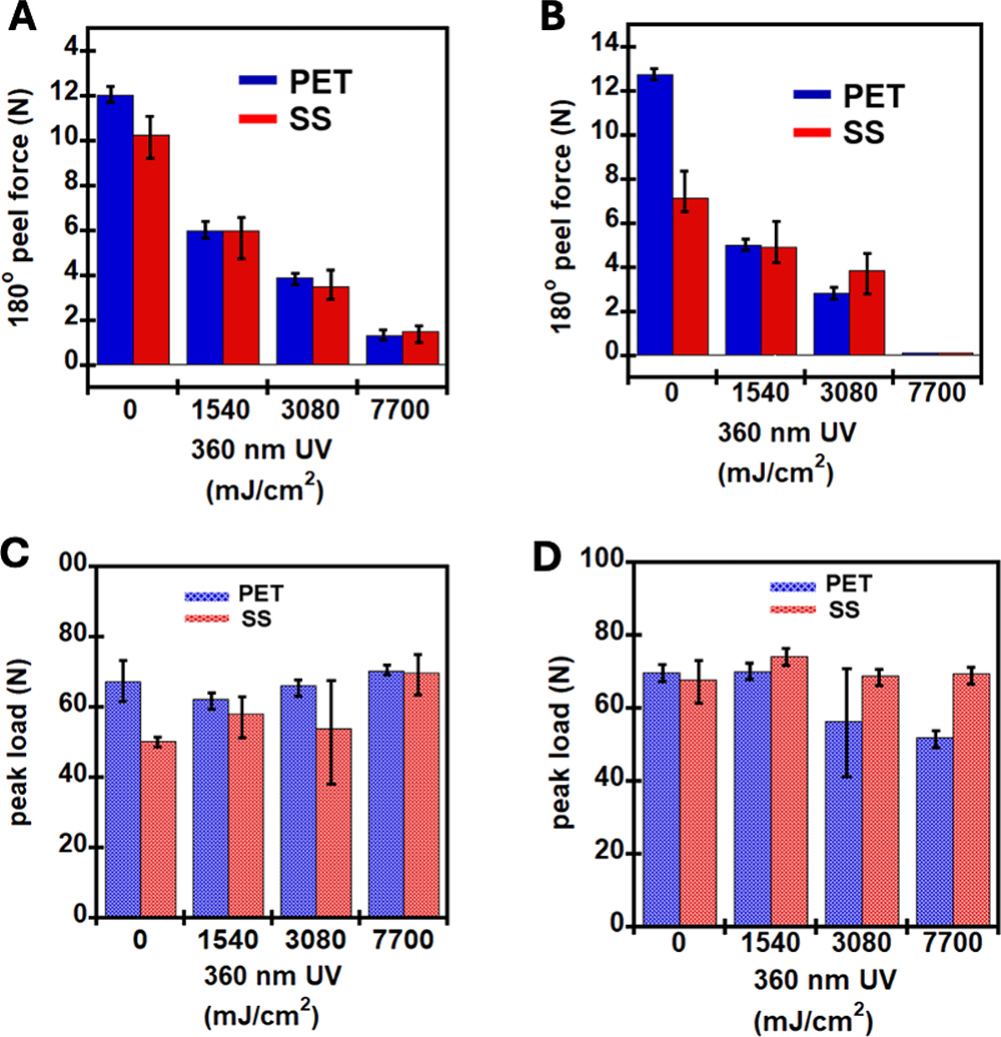
180° adhesion peel test and effect of UV intensity on (A) poly­(BA_91.5_-Al_0.5_-AA_3_-CM_5_) and (B) poly­(BA_87.5_-Al_0.5_-AA_3_-CM_10_) and dynamic shear plots for (C) poly­(BA_91.5_-Al_0.5_-AA_3_-CM_5_) and (D) poly­(BA_87.5_-Al_0.5_-AA_3_-CM_10_).

For both 5% and 10% CM containing PSAs, however, the dynamic shears remained unchanged or changed ever so slightly, regardless of the substrate panel type, as shown in Figure 8C,D and Table S6. This is not surprising considering that the dynamic shear test characterizes the cohesive nature of the PSA, which reflects its elasticity. With UV exposure, there occurs an increase in the cross-linking consuming the CM species and buildup of elasticity, i.e., *G*′, as also seen in our rheology data. Within the confines on the dynamic shear test geometry, this characterization seems not as sensitive to reflect the changes in the elasticity buildup due to CM cross-linking. This observation also shows an important finding that debonding is subject to deformation geometry or direction. For our system, debonding is readily possible in peel direction than in shear direction or force fields. This ability of debonding directionality or anisotropy can have some creative practical implications.

In addition to the increased *T*_g_ and *G*′ upon UV irradiation, volume shrinkage is an important parameter, which helps in achieving efficient DoD because the shrinkage could lead to interfacial cracks causing the in situ DoD. The effect of photoirradiation on the volume of the PSAs is shown in Table 1 and Figure 8. After exposure to 360 nm UV, a significant reduction in a volume of ∼17% of the original volume prior to UV exposure was observed. As seen in both Figure 9A,B, irrespective of the nature of the cross-linker, there is a notable amount of volume reduction in the synthesized PSAs after UV irradiation. For both networks cross-linked with either EGDMA or Al-AA, lower %CM containing networks resulted in smaller volume reduction (∼3.5–9.2%), while networks with more CM monomer (10%) gave relatively higher volume reduction (∼9.6 to 6.6%). This is consistent with earlier experiments where higher CM loading resulted in significant effects compared to networks with a lower CM monomer.

**Figure 9:**
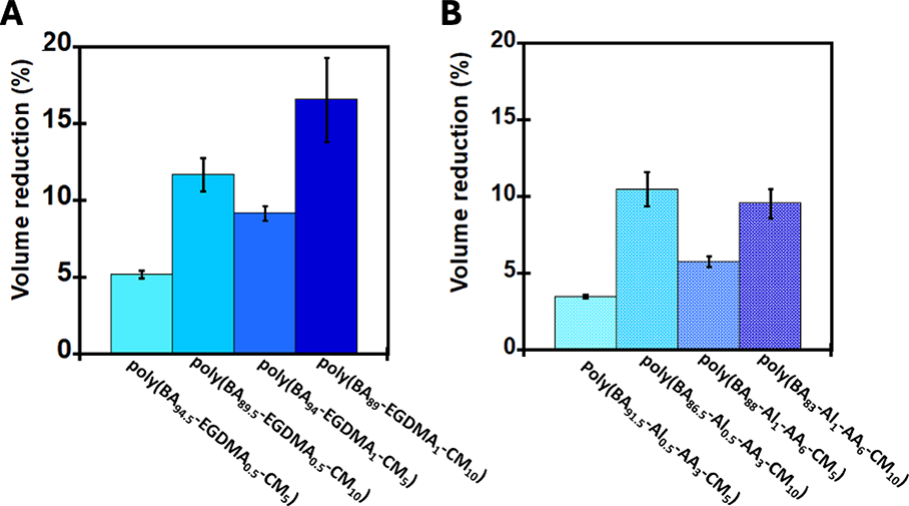
Volume change of the photoresponsive PSAs after 360 nm UV irradiation for 12 h. (A) EGDMA cross-linked PSAs and (B) Al-AA cross-linked PSAs.

## Conclusion

In conclusion, we designed photoresponsive coumarin PSAs with excellent DoD properties by using free radical polymerization techniques. Networks with dynamic Al-AA linkers poly­(BA-Al-AA-CM) and static EGDMA poly­(BA-EGDMA-CM) cross-linkers were developed. The photoresponsiveness of the networks was independent of the nature of the cross-linker but dependent on the quantity of the light sensitive coumarin monomer. Networks with a more CM moiety showed larger increases in *T*_g_, moduli, and volume reduction after UV irradiation compared to networks with a lower CM monomer. In addition, poly­(BA-Al-AA-CM) systems showed improved adhesive strength compared to poly­(BA-EGDMA-CM) due to their dynamic cross-links that can allow better wetting of the substrate. In all cases, after UV irradiation, essentially complete debonding was observed. The results of this study demonstrate the promising properties of simple photoresponsive dual cross-linked coumarin-based materials for efficient DD PSAs in industrial processes.

## Experimental Section

### Materials

All starting materials, solvents, and reagents were purchased from commercial sources and used directly without further purification unless otherwise stated. Thermal initiated 2,2′-azobis­(4-methoxy-2,4-dimethylvaleronitrile) (V-70) was obtained from Fujifilm Wako. Butyl acrylate (BA) and ethylene glycol dimethacrylate (EGDMA) were obtained from ACROS Organics. Aluminum acetyl acetonate (AAA) was obtained from TCI. 7-Hydroxy coumarin was obtained from Carbosynth. 2-Oxo-2*H*-chromen-7-yl acrylate (coumarin monomer) was synthesized as outlined in the literature.^33^

### Monomer Synthesis

#### Synthesis of 2-Oxo-2*H*-chromen-7-yl Acrylate (Coumarin Monomer)

EDC (29.25g, 0.1870 mol) and 150 mL of DCM were added into a 500 mL round-bottom flask with a stir bar. The flask was put in an ice bath, and 7-hydroxy coumarin (17.68g, 0.1210 mol) was added to the flask. Next, while stirring, AA (11.17g, 0.1550 mol) was added to the flask. Lastly, DMAP (0.95g, 0.0078 mol) was added. The flask was then sealed and stirred for 48 h. Once the reaction finished, the reaction mixture was washed with 0.2 M HCl (3×), brine (3×), saturated NaHCO_3_ (3×), water (1×), and finally brine (3×). The separated organic phase was dried in MgSO_4_, and the solution was evaporated in vacuum to yield coumarin monomer 2-oxo-2*H*-chromen-7-yl acrylate (CM) as a slightly pinkish solid (18.6 g 0.0864 mol, 72% yield).

#### ^1^H NMR (CDCl_3_, 500 MHz)

δ ppm 7.70 (d, J = 9.56 Hz, 1H), 7.50 (d, J = 8.49 Hz, 1H), 7.17 (d, J = 2.25 Hz, 1H), 7.10 (dd, J = 8.56 Hz, 2.17 Hz, 1H), 6.66 (dd, J = 17.29 Hz, 1.02 Hz, 1H), 6.41 (d, J = 9.59 Hz, 1H), 6.33 (dd, J = 17.39 Hz, 10.41 Hz, 1H), 6.09 (dd, J = 10.43 Hz, 1.00 Hz, 1H).

### Polymer Synthesis

#### Typical Synthesis of Poly­(BA-EGDMA-CM) Materials

Butyl acrylate (BA) (4 g), 2,2′-azobis­(4-methoxy-2,4-dimethylvaleronitrile) (V-70) (40 mg), and *N*,*N*-dimethylformamide (DMF) (6 mL) were added in a glass vial. In that mixture, EGDMA (0.02 and 0.04 g) and coumarin monomer (CM) (5% of BA and 10% of BA) were then added to synthesize poly­(BA-0.5%EGDMA-5%CM), poly­(BA-0.5%EGDMA-10%CM), poly­(BA-1%EGDMA-5%CM), and poly­(BA-1%EGDMA-10%CM) materials. The mixture was sonicated for 10 min for proper mixing/dissolution and then transferred to a Teflon mold for polymerization. The free radical polymerization was carried out at 35 °C for 3 h. After the polymerization, the cross-linked material was removed from the mold and allowed to dry for 3 days at an ambient condition. The materials were further dried overnight in a vacuum oven at 40 °C. A similar control was made following this procedure with no added CM monomer using 0.5% EGDMA.

#### Typical Synthesis of Poly­(BA-AAA-AA-CM) Materials

Butyl acrylate (BA) (4 g), 2,2′-azobis­(4-methoxy-2,4-dimethylvaleronitrile) (V-70) (40 mg), and *N*,*N*-dimethylformamide (DMF) (8 mL) were added in a glass vial. In that mixture, acrylic acid (3% and 6% by weight) and coumarin monomer (CM) (5% and 10% by weight) were then added to synthesize poly­(BA-AAA-AA-CM) materials. The mixture was sonicated for 10 min for proper mixing/dissolution and then transferred to a Teflon mold for polymerization. After about an hour, AAA was added to the mixture (0.5% or 1% by weight), made from a solution of aluminum acetyl acetonate (AAA):excess acetyl acetonate:toluene in 1:3:9 by weight. The free radical polymerization was carried out at 35 °C for 3 h. After the polymerization, the cross-linked material was removed from the mold and allowed to dry for 3 days at an ambient condition. The materials were further dried overnight in a vacuum oven at 40 °C.

### Characterization

#### Swelling Ratio

The dried PSAs (70–180 mg) were weighed and soaked in excess THF at room temperature (22 °C) for 48 h. Subsequently, the swollen polymers were weighed, and the swelling ratio was calculated as stated in eq 1.

$$\mathrm{swelling}\;\mathrm{ratio}=\frac{W_{s}-W_{d}}{W_{d}}$$ 1

where *W*_s_ is the weight of the swollen sample and *W*_d_ is the weight of the dried sample.

#### Gel Fraction

The dried PSAs (70–180 mg) were weighed and soaked in excess THF at room temperature (22 °C) for 5 days. After 5 days, the solvent was carefully drained and allowed to stand for 5 days. Subsequently, the swollen polymers were weighed, and the gel fraction was calculated as stated in eq 2.

$$\mathrm{swelling}\;\mathrm{ratio}=\frac{W_{f}}{W_{i}}$$ 2

where *W*_f_ is the final dried weight and *W*_i_ is the initial weight.

#### Photoreactor Intensity Measurement

The power intensity of the photoreactor was determined using a wavelength-tunable Thorlabs PM100A meter with a S120VC silicon photodiode. Intensity was calculated by dividing the measured intensity by the area of the 0.9 cm diameter aperture in the detector.

#### Rheology

Rheological frequency sweep experiments were carried out using a TA Instruments (New Castle, DE) Discovery HR-1 rheometer. A 20 mm cross-hatched plate geometry was used for all the experiments. In all cases, rheology disks were synthesized in a 20 mm diameter circular Teflon mold. Frequency sweep experiments were carried out from 0.1 to 500 Hz angular frequency with 0.5% applied strain at 25 °C.

Time sweep rheology under photochemical irradiation was performed on a TA Instruments (New Castle, DE) Discovery HR-2 rheometer with a 20 mm parallel plate geometry at 10 rad/s and 1% strain at 40 °C. Photorheology was performed under irradiation from a OmniCure S2000 High pressure Hg vapor short arc lamp using a 320–500 nm filter.

#### Differential Scanning Calorimetry (DSC)

All glass transition temperatures (*T*_g_) were obtained by using a TA Instruments DSC Q2000. The data was obtained in a heat–cool–heat cycle ranging from −60 to 100 °C with a 10 °C/min heating rate with a total of three heating cycles. To remove thermal history, data from the first heating cycle was not analyzed. Data from the second heating cycle was used to plot the curve, and data from the second and third cycle were used to determine the mean and standard deviation of the *T*_g_.

#### Lap Shear

Uniaxial tensile testing experiments were carried out using PET, glass, and stainless steel as the substrate. The network materials were sandwiched between two substrates of the same materials, and they were attached to an Instron 3344 universal testing system equipped with a 2000 N load cell. The extension rate was 5 mm/s, and data were collected until the material failed.

#### PSA Sample Preparation for Peel and Dynamic Shear Tests

After completion of polymerization, AAA solution was added to the polymer in stipulated quantities. Then, the polymer solution was directly coated onto a 50 um PET film facestock and dried at 120 °C for 10 min. The dried PSA was equilibrated at 22 ± 1 °C and 50 ± 5% RH for at least 24 h before adhesion testing.

#### Substrate Panel Preparation

SS panels were rinsed with MEK and thoroughly wiped using Kimwipes. PET panels were first rinsed with heptane, wiped with a Kimwipe, rinsed with IPA, and finally allowed to dry off. Both the cleaned SS and PET panels were conditioned at 22 ± 1 °C and 50 ± 5% RH for at least 1 h before adhesion testing.

#### 180° Peel Adhesion

180° peel adhesion was measured over a PSA strip on PET film face that is 1 in. wide and at least 6 in. in length. The strip was attached to respective substrate panels rolled over by a 4.5 lb rolled at 24 in./min for two cycles. Upon this bonding step, the dwell time was 15 min before the peel adhesion was measured in 180° geometry at 12 in./min speed using an MTS testing machine.

#### Dynamic Shear Test

Dynamic shear for PSA strips was measured over 0.5 in. wide samples applied over 0.5 in. width on corresponding substrates. Thus, the deforming area was 0.25 in.^2^. The strip was attached to respective substrate panels rolled over by a 4.5 lb rolled at 24″/min for two cycles. Upon this bonding step, the dwell time was 15 min before the shear adhesion was measured at 0.25 in./min speed using an MTS testing machine.

#### Debonding of Adhesives

The adhesive materials were sandwiched and bonded between two PET substrates, and a roller of 4.5 lb was rolled back and forth twice on the PET substrates containing the adhesive materials to obtain a firm bond between the adhesive material and the PET substrates. The adhesives were debonded from the PET substrate using a photoreactor with UV lamps with a peak near 360 nm^55^ with an intensity of 4.8 ± 0.5 mW/cm^2^, measured as a straight-line intensity. To obtain 2 + 2 cycloaddition cross-linked polymers using UV light, the adhesive materials were irradiated with UV light of approximately 360 nm at ambient temperature and pressure. The distance between the PET substrate and the light source was 1.57 in. and was kept constant until the experiment was ended after 12 h of UV irradiation. The resultant PET substrate was debonded from adhesive material. The reversibility of the cross-linked polymers was carried out under UV light of 250 nm at ambient temperature and pressure for 12 h with the same experimental set up as the 360 nm UV.

#### Volume Reduction

Rectangular molds were used to prepare photoresponsive PSAs. The PSAs were allowed to dry in the oven overnight and at room temperature for 24 h. The dimensions of the rectangular adhesive materials were measured before and after UV exposure, and the volume reduction was calculated using the dimensions.

## Supplementary Material











## Notes

Adhesive test setup with photoresponsive coumarin monomer before UV exposure (MP4)
Adhesive test setup with photoresponsive coumarin monomer after UV exposure (MP4)
PSA with photoresponsive coumarin monomer before UV exposure (MP4)
PSA with photoresponsive coumarin monomer after UV exposure (MP4)
Supplemental rheological data, supplemental adhesion data, swelling and gel fraction data, infrared spectroscopy data, and typical specimen data (PDF)
