# Supplementary material for: Photoresponsive Polymers for Debonding-on-Demand Pressure-Sensitive Adhesives
Source: ACS Appl Mater Interfaces. 2026 Mar 26;18(13):19599–609. doi: 10.1021/acsami.5c25897 (PMC13067236; doi:10.1021/acsami.5c25897)
Supplement: Supplementary file 5 [file am5c25897_si_005.pdf]

Supporting Information: Photoresponsive Polymers for Debonding-on-Demand Pressure Sensitive Adhesives

Ibrahim O. Raji[a], Nishad Dhopatkar[b], Thomas G. Ribelli[b], Madison Saldanha[a], Michele Fromel,[b] Eric Bartholomew[b] and Dominik Konkolewicz*[a]
[a] Department of Chemistry and Biochemistry, Miami University, 651 E High St, Oxford, OH 45056, USA.
[b] Avery Dennison, 171 Draketown Rd, Mill Hall, PA 17751, USA
E-mail: d.konkolewicz@miamiOH.edu
*Corresponding author

Supplemental Data

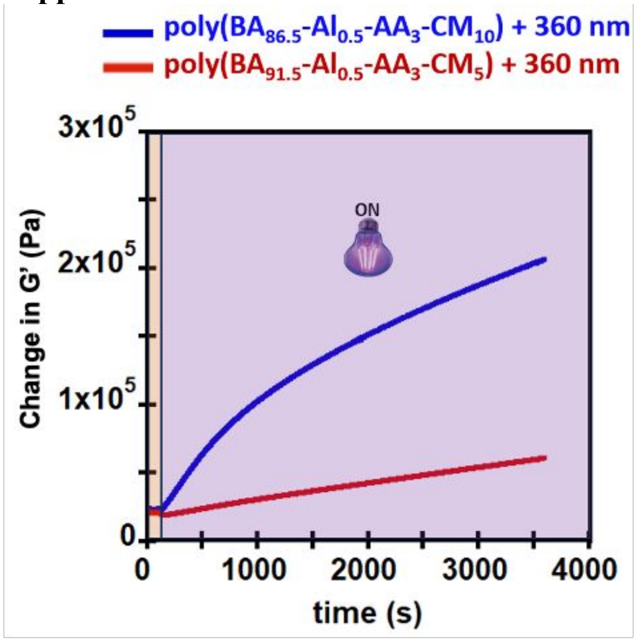

Figure S1: Photorheometry time sweeps of the light-responsive PSAs for poly($BA_{91.5}$-$Al_{0.5}$-AA_3-CM_5) and poly($BA_{86.5}$-$Al_{0.5}$-AA_3-CM_{10}).

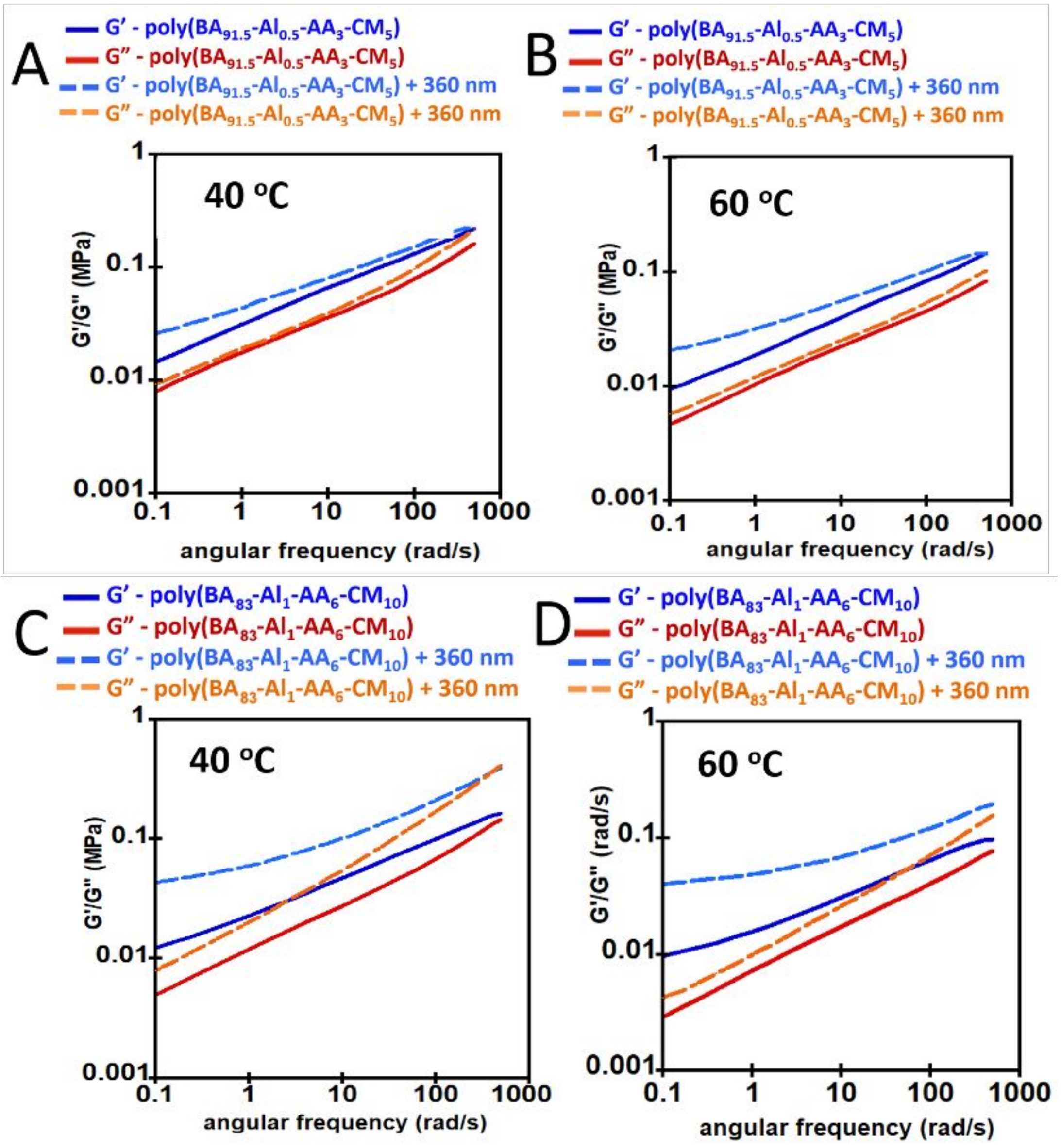

Figure S2: Frequency sweep plots before and after 360 nm UV for (a) poly(BA_{88}-Al_1-AA_6-CM_5), at 40 ^{o}C, (b) 60 ^{o}C, (c) poly(BA_{83}-Al_1-AA_6-CM_{10}), at 40 ^{o}C and, (d) 60 ^{o}C

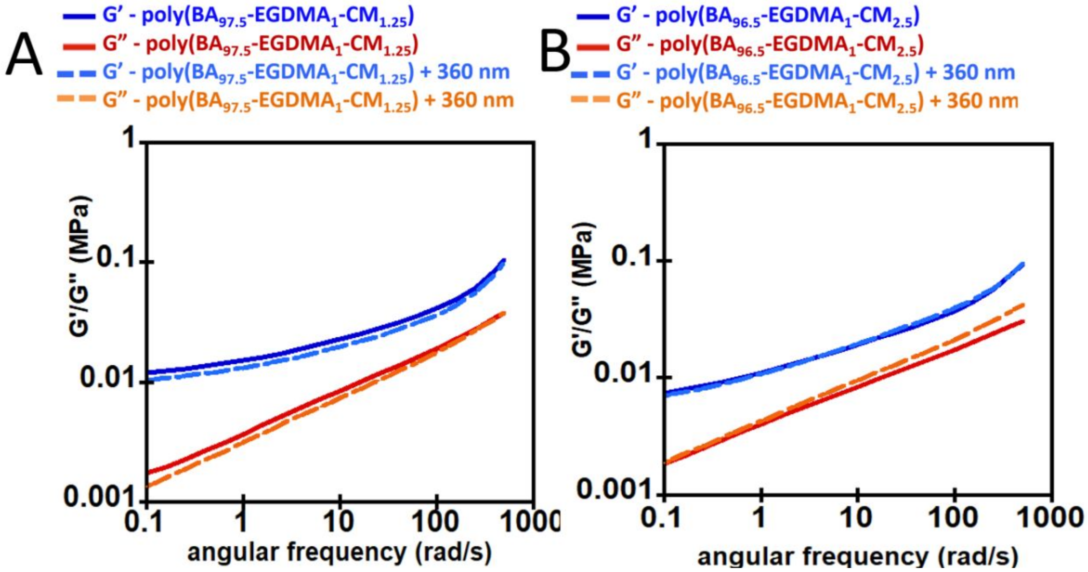

Figure S3: Frequency sweep plots before and after 360 nm UV for (a) poly($BA_{97.5}$-$EGDMA_1$-$CM_{1.25}$) and (b) poly($BA_{96.5}$-$EGDMA_1$-$CM_{2.5}$).

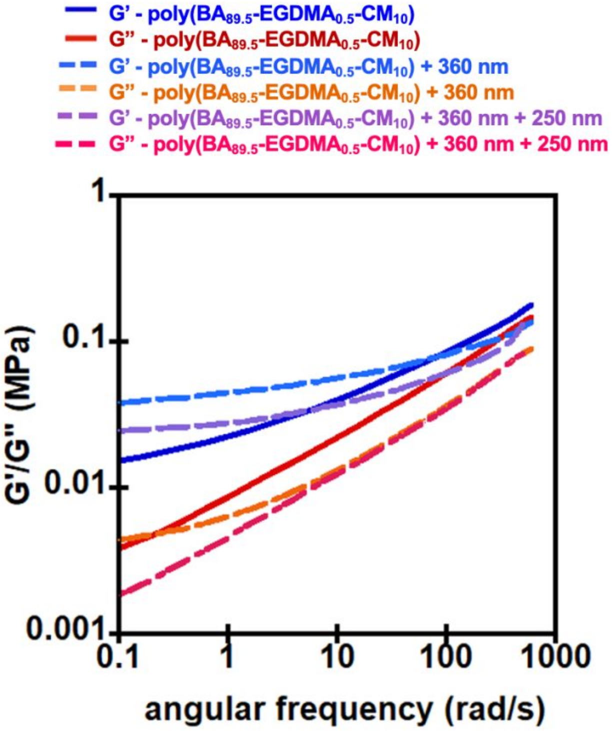

Figure S4: Frequency sweep plots before, after 360 nm and 250 nm UV poly($BA_{89.5}$-$EGDMA_{0.5}$-CM_{10})

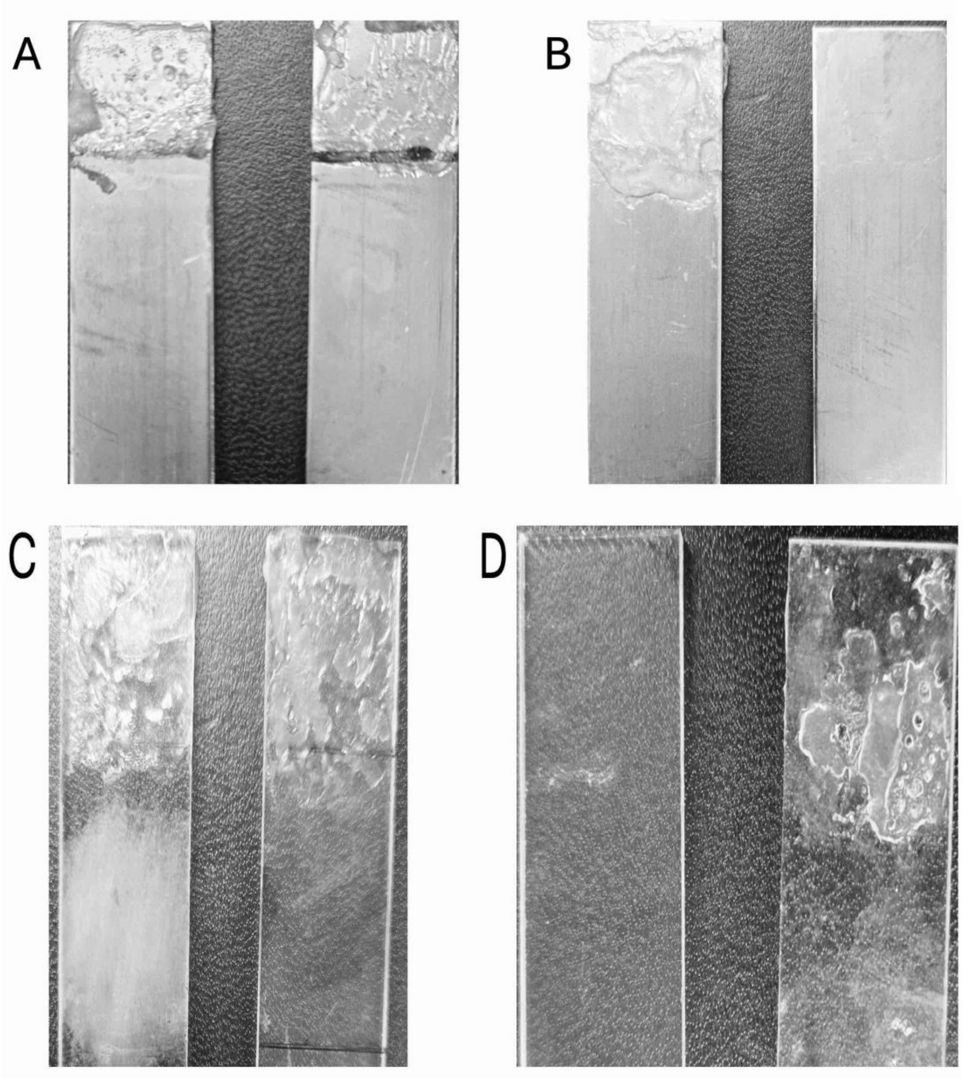

Figure S5: Failure modes for (a) poly(BA-Al-AA-CM) PSA with SS substrate (cohesive failure) (b) poly(BA-EGDMA-CM) PSA with SS substrate (adhesive failure) (c) poly(BA-Al-AA-CM) PSA with PET substrate (cohesive failure) (d) poly(BA-EGDMA-CM) PSA with PET substrate (adhesive failure).

Table S1: Swelling ratio and gel fraction of the synthesized PSAs before and after 360 nm UV

Networks	Swelling Ratio (before 360 nm UV)	Swelling Ratio (after 360 nm UV)	Gel Fraction (before 360 nm UV)	Gel Fraction (after 360 nm UV)
poly($BA_{94.5}$-$EGDMA_{0.5}$-CM_5)	13.6±0.8	13.0±0.5	99.88±0.02	99.85±0.01
poly($BA_{84.5}$-$EGDMA_{0.5}$-CM_{10})	12.5±0.6	9.7±0.4	99.7±0.2	95±4
poly(BA_{94}-$EGDMA_1$-CM_5)	13.2±0.4	12.2±0.3	99.7±0.1	97.2±0.3
poly(BA_{84}-$EGDMA_1$-CM_{10})	12±1	9.0±0.3	99.84±0.01	96.95±0.02
poly($BA_{91.5}$-$Al_{0.5}$-AA_3-CM_5)	15±2	12.6±0.5	99.98±0.0	96.92±0.01
poly($BA_{86.5}$-$Al_{0.5}$-AA_3-CM_{10})	13±1	10.7±0.5	99.98±0.0	99±2
poly(BA_{88}-Al_1-AA_6-CM_5)	12.3±0.4	11.1±0.6	99.82±0.06	99.90±0.00
poly(BA_{83}-Al_1-AA_6-CM_{10})	10.0±0.6	7.8±0.2	99.8±0.6	99.98±0.01

Table S2: Adhesive strength measured by lap shear with SS substrate.

Networks	S_{shear} (kPa) (SS)[d]
poly($BA_{94.5}$-$EGDMA_{0.5}$-CM_5)	4±2
poly($BA_{89.5}$-$EGDMA_{0.5}$-CM_{10})	6±1
poly(BA_{94}-$EGDMA_1$-CM_5)	10±0.1
poly(BA_{89}-$EGDMA_1$-CM_{10})	0.9±0.4
poly($BA_{91.5}$-$Al_{0.5}$-AA_3-CM_5)	19±4
poly($BA_{86.5}$-$Al_{0.5}$-AA_3-CM_{10})	11±1
poly(BA_{88}-Al_1-AA_6-CM_5)	17±0.7
poly(BA_{83}-Al_1-AA_6-CM_{10})	12±2

Table S3: Adhesive strength measured by lap shear with PET substrate after 360 nm and 250 nm UV.

Networks	Initial adhesive strength (kPa)	Adhesive strength after 360 nm (kPa)	Adhesive strength after 250 nm (kPa)
poly($BA_{89.5}$-$EGDMA_{0.5}$-CM_{10})	24±4	0	6±1

Table S4: Adhesive strength measured by lap shear with PET substrate after soaking in water for 48 h

Networks	S_{shear} (kPa) (PET) 0 h in H_2O	S_{shear} (kPa) (PET) 48 h in H_2O
poly($BA_{94.5}$-$EGDMA_{0.5}$-CM_5)	24±4	25±0.5
poly($BA_{91.5}$-$Al_{0.5}$-AA_3-CM_5)	22±3	14±3

Table S5: Adhesive strength measured by lap shear with PET and Glass substrates

Networks	S_{shear} (kPa) PET(substrate)	S_{shear} (kPa) Glass (substrate)

poly(BA$_{94.5}$-EGDMA$_{0.5}$-CM$_{5}$)	24±4	8±0.5
poly(BA$_{91.5}$-Al$_{0.5}$-AA$_{3}$-CM$_{5}$)	22±3	5±0.7

Table S6: Adhesive strength measured by 180° adhesive peel and dynamic shear for 5% CM and 10 % CM with PET and Glass substrates

360 nm UV intensity (mJ/cm^{2})	180° peel (N) 5% CM PET	180° peel (N) 5% CM SS	180° peel (N) 10%CM PET	180° peel (N) 10% CM SS	Dynamic Shear (N) 5% CM PET	Dynamic Shear (N) 5% CM SS	Dynamic Shear (N) 10% CM PET	Dynamic Shear (N) 10% CM SS
0	12.0±0.5	10.2±0.5	12.7±0.1	7.1±0.9	67±6	50±1	70±3	68±4
1540	6.0±0.4	6.0±0.5	5.0±0.2	5±1	62±3	58±7	70±2	74±2
3080	4±1	3.5±0.5	2.8±0.1	3.8±0.4	66±3	50±10	60±10	69±3
7700	1.3±0.1	1.5±0.3	0.1±0.0	0.1±0.0	70±1	70±4	52±3	70±2

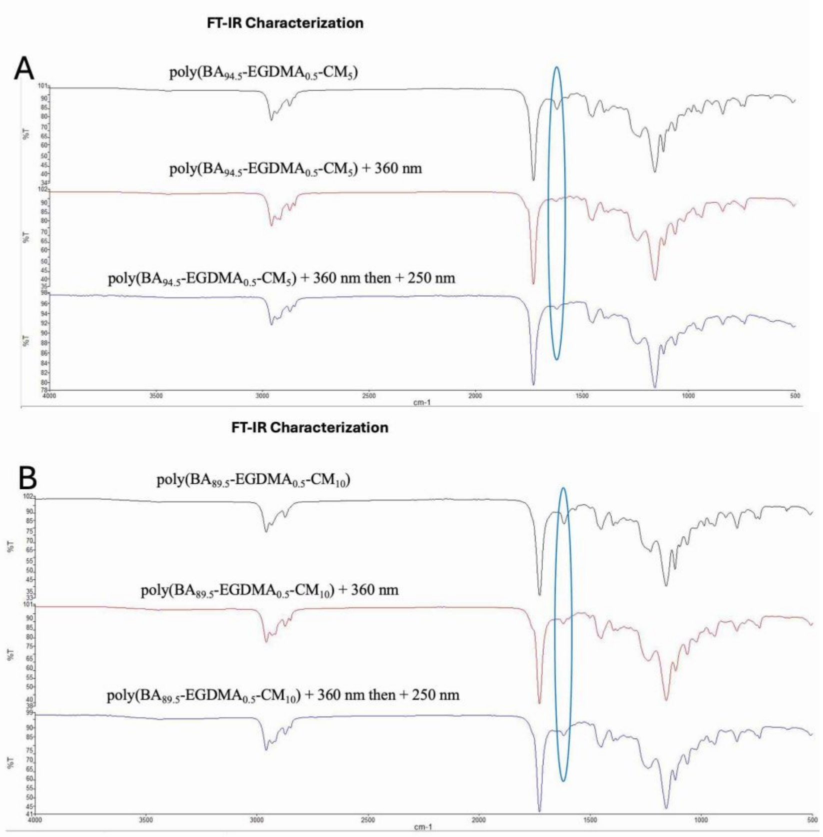

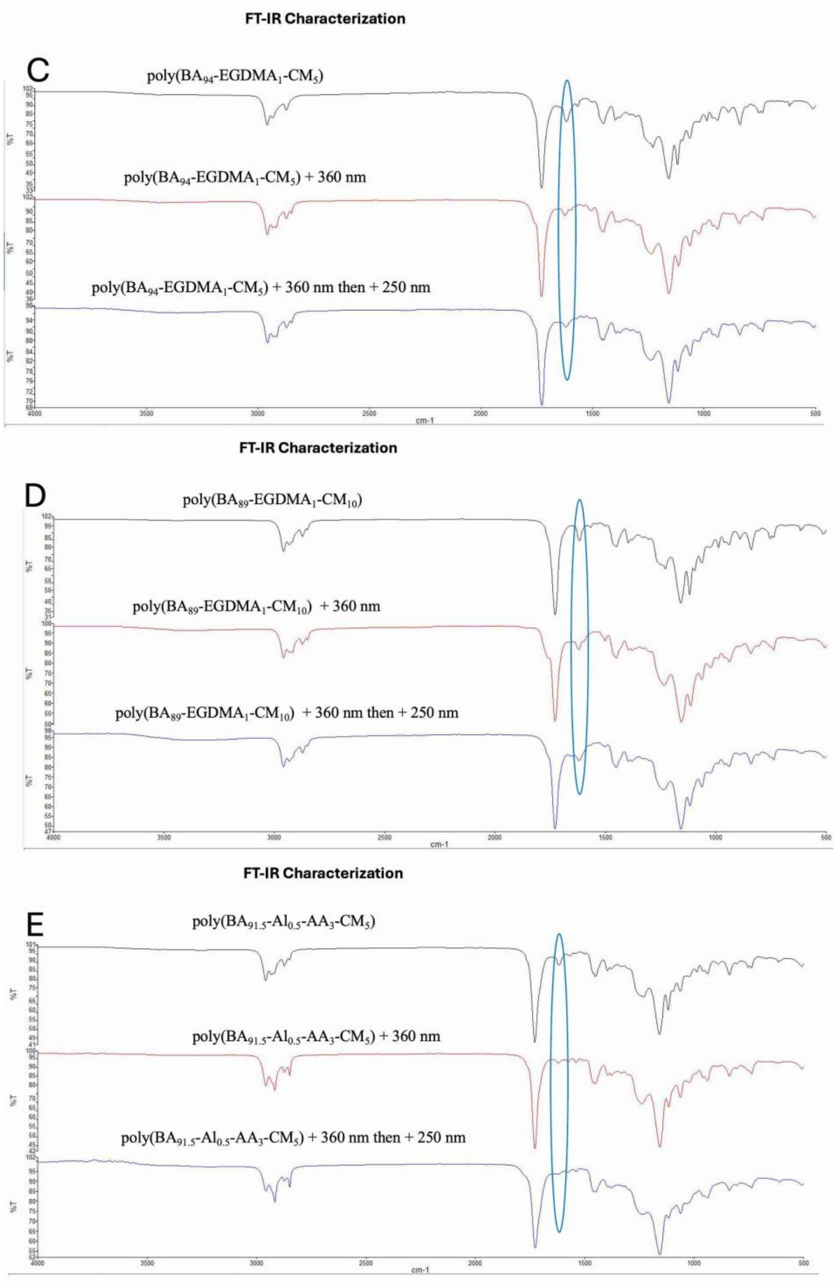
FT-IR Characterization
C
poly(BA_{94}-$EGDMA_1$-CM_5)
poly(BA_{94}-$EGDMA_1$-CM_5) + 360 nm
poly(BA_{94}-$EGDMA_1$-CM_5) + 360 nm then + 250 nm
%T
cm-1
4000
3500
3000
2500
2000
1500
1000
500
FT-IR Characterization
D
poly(BA_{89}-$EGDMA_1$-CM_{10})
poly(BA_{89}-$EGDMA_1$-CM_{10}) + 360 nm
poly(BA_{89}-$EGDMA_1$-CM_{10}) + 360 nm then + 250 nm
%T
cm-1
4000
3500
3000
2500
2000
1500
1000
500
FT-IR Characterization
E
poly($BA_{91.5}$-$Al_{0.5}$-AA_3-CM_5)
poly($BA_{91.5}$-$Al_{0.5}$-AA_3-CM_5) + 360 nm
poly($BA_{91.5}$-$Al_{0.5}$-AA_3-CM_5) + 360 nm then + 250 nm
%T
cm-1
4000
3500
3000
2500
2000
1500
1000
500

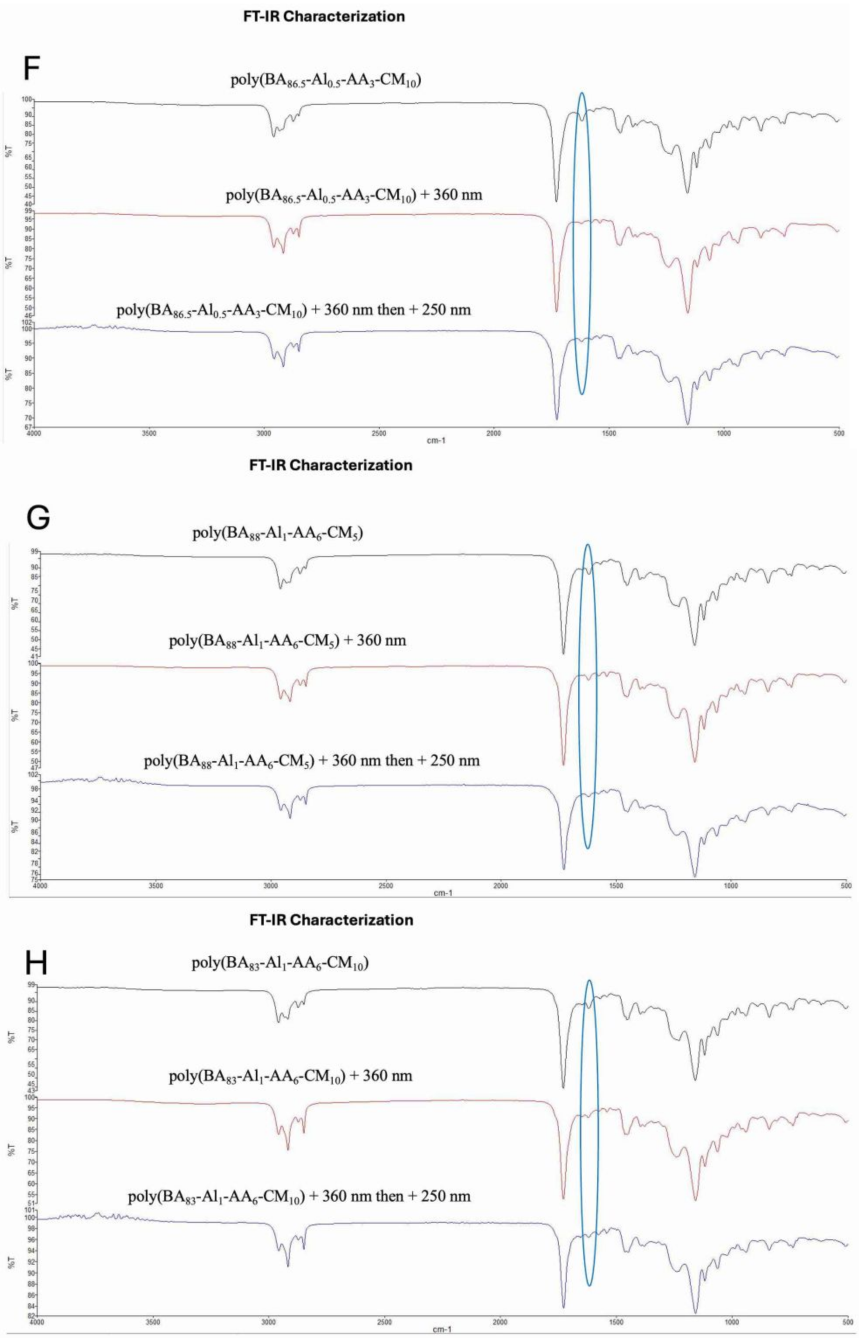

Figure S6: FT-IR characterization of the coumarin-based PSAs (a) poly($BA_{94.5}$-$EGDMA_{0.5}$-CM_5), (b) poly($BA_{84.5}$-$EGDMA_{0.5}$-CM_{10}), (c) poly(BA_{94}-$EGDMA_1$-CM_5), (d) poly(BA_{84}-$EGDMA_1$-CM_{10}), (e) poly($BA_{91.5}$-$Al_{0.5}$-AA_3-CM_5), (f) poly($BA_{86.5}$-$Al_{0.5}$-AA_3-CM_{10}), (g) poly(BA_{88}-Al_1-AA_6-CM_5), and (h) poly(BA_{83}-Al_1-AA_6-CM_{10})

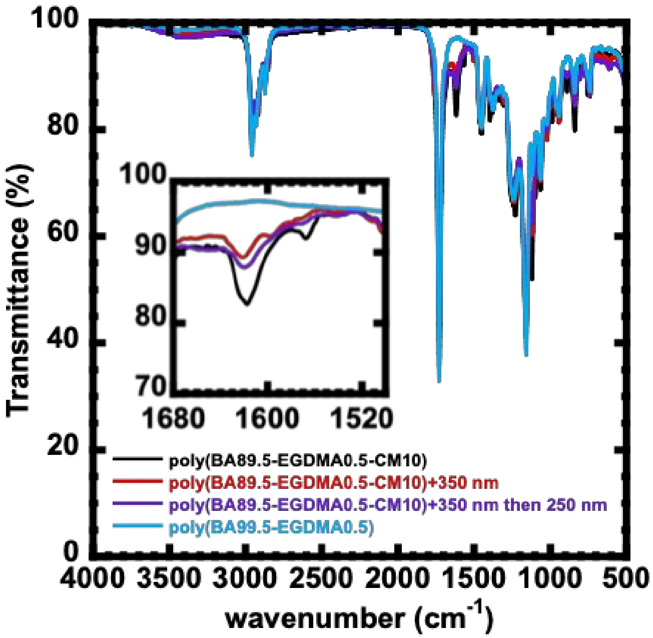

Figure S7. FTIR data for poly($BA_{89.5}$-$EGDMA_{0.5}$-CM_{10}) before after synthesis (black), after exposure to 360 nm UV radiation (red), after subsequent exposure to (250 nm UV). Comparison against a control poly($BA_{99.5}$-$EGDMA_{0.5}$) shows the absence of vinyl signals (~1620 cm^{-1}) without CM.

Molar mass between crosslinks was estimated using the formula below[3]:

$$M_c = \frac{\rho RT}{G'_{plat}} \qquad \text{(S1)}$$

Where ρ is the bulk density, R is the universal gas constant, T is the absolute temperature, and G'_{plat} is the plateau modulus. Here the storage modulus G' at 0.01 rad/s is used to estimate the plateau modulus. Estimates of molar masses between crosslinks is given in Table S7.

Table S7: Estimates of molar mass between crosslinks, using G' at 0.01 rad/s as the estimate of G'plat in Eq S1. Bulk density was assumed to be ρ = 1080 kg/m^3.[4]

System	G' before 360 nm (kPa)	G' after 360 nm (kPa)	M_c (before UV) (Da)	M_c (after UV) (Da)
EGDMA 0.5% CM 5%	8.93E+00	1.99E+01	3.0×10^5	1.3×10^5
EGDMA 1% CM 5%	1.52E+01	3.81E+01	1.8×10^5	7.0×10^4
EGDMA 0.5% CM 10%	1.28E+01	2.45E+01	2.1×10^5	1.1×10^5
EGDMA 1% CM 10%	1.41E+01	5.27E+01	1.9×10^5	5.1×10^4
AAA 0.5% CM 5%	5.22E+00	1.09E+01	5.1×10^5	2.5×10^5
AAA 1% CM 5%	8.29E+00	2.96E+01	3.2×10^5	9.0×10^4
AAA 0.5% CM 10%	1.30E+01	1.88E+01	2.1×10^5	1.4×10^5
AAA 1% CM 10%	1.55E+01	3.61E+01	1.72×10^5	7.4×10^4

Table S8: Typical Dimensions of samples and adhesives used in different geometries.

Geometry	Experiment Type	Width (mm)	Length (mm)	Diameter (mm)	Thickness (mm)
Circular Disc	Rheology (frequency sweep)	N/A	N/A	19.7±0.3	3.1±0.3
Rectangular Prism	Lap Shear (PET substrate)	20.07±0.05	22±1	N/A	3.0±0.3
Rectangular Prism	Lap Shear (SS substrate)	19.93±0.06	19±1	N/A	3.0±0.3
Rectangular Prism	Lap Shear (glass substrate)	21.2±0.3	15.2±0.2	N/A	3.0±0.3
Rectangular Prism	Peel Test (film)	25.0±0.1	N/A	N/A	0.035±0.003

References

(1) Chakma, P.; Wanasinghe, S. V; Morley, C. N.; Francesconi, S. C.; Saito, K.; Sparks, J. L.; Konkolewicz, D. Heat- and Light-Responsive Materials Through Pairing Dynamic Thiol–Michael and Coumarin Chemistry. *Macromol. Rapid Commun.* **2021**, *42* (18), 2100070. https://doi.org/https://doi.org/10.1002/marc.202100070.

(2) Dowdy, J. C.; Sayre, R. M. Photobiological Safety Evaluation of UV Nail Lamps. *Photochem. Photobiol.* **2013**, *89* (4), 961–967. https://doi.org/https://doi.org/10.1111/php.12075.

(3) Rajawasam, C. W. H.; Dodo, O. J.; Weerasinghe, M. A. S. N.; Raji, I. O.; Wanasinghe, S. V.; Konkolewicz, D.; De Alwis Watuthanthrige, N. Educational Series: Characterizing Crosslinked Polymer Networks. *Polymer Chemistry*. Royal Society of Chemistry December 19, 2023, pp 219–247. https://doi.org/10.1039/d3py00914a.

(4) SCIENTIFIC POLYMER PRODUCTS INC. Poly(n-Butyl Acrylate). *https://scipoly.com/wp-content/uploads/2020/09/234-tds-1.pdf.*
